# Targeting CDK2 Confers Vulnerability to Lenvatinib Via Driving Senescence in Anaplastic Thyroid Cancer

**DOI:** 10.1002/advs.202413514

**Published:** 2024-12-24

**Authors:** Ben Ma, Youzhou Sang, Xiaoxue Du, Yanzhi Zhang, Min Yin, Weibo Xu, Wanlin Liu, Jiayi Lu, Qing Guan, Yunjun Wang, Tian Liao, Yuting Wang, Jun Xiang, Rongliang Shi, Ning Qu, Qinghai Ji, Jiwei Zhang, Dongmei Ji, Yu Wang

**Affiliations:** ^1^ Department of Head and Neck Surgery Fudan University Shanghai Cancer Center Shanghai 200032 P. R. China; ^2^ Department of Oncology Shanghai Medical College Fudan University Shanghai 200032 P. R. China; ^3^ Department of Medical Oncology Fudan University Shanghai Cancer Center Shanghai 200032 P. R. China; ^4^ The MOE Key Laboratory for Standardization of Chinese Medicines Institute of Chinese Materia Medica Shanghai University of Traditional Chinese Medicine Shanghai 201203 P. R. China

**Keywords:** ATC, CDK2, Dinaciclib, FBW7, PDX, PF‐07104091

## Abstract

Anaplastic thyroid cancer (ATC) is the most lethal tumor arising from thyroid follicular epithelium. Lenvatinib is an off‐label use option for ATC patients in many countries but an approved prescription in Japan. However, lenvatinib resistance is a substantial clinical challenge. Clinical ATC samples including lenvatinib‐resistant tumors are used to build patient‐derived cells and patient‐derived xenografts. High‐throughput drug screening and synergy analyses are performed to identify an effective combination partner for lenvatinib. Cellular functions are detected by cell senescence, apoptosis, cell cycle, cell viability and colony formation assays. CDK2 inhibition showed the significant synthetic lethality with lenvatinib via inhibiting G1/S transition and inducing cell senescence in ATC. High expression of CDK2 is associated with lenvatinib resistance and poor clinical outcomes of ATC patients. Lenvatinib increased protein expression of CDK2 in lenvatinib‐resistant ATC cells. Mechanistically, lenvatinib inhibited protein degradation of CDK2 via reducing CDK2's interaction with the RACK1‐FBW7 complex, which is involved in ubiquitination and subsequent proteasomal degradation of CDK2. Combination of CDK2 inhibitors in clinical trials (Dinaciclib or PF‐07104091) and lenvatinib markedly suppressed growth of xenograft tumors from the lenvatinib‐resistant patient. The findings support the combination therapy strategy of lenvatinib and CDK2 inhibitor for lenvatinib‐resistant ATC patients with high CDK2 expression.

## Graphical abstract

Anaplastic thyroid cancer (ATC) is the most lethal tumor arising from thyroid follicular epithelium. Lenvatinib is an off‐label use option for ATC patients in many countries but an approved prescription in Japan. However, lenvatinib resistance is a substantial clinical challenge. The findings support the combination therapy strategy of lenvatinib and CDK2 inhibitor for lenvatinib‐resistant ATC patients with high CDK2 expression.

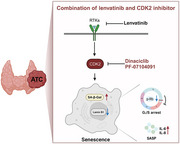

## 1 Introduction

Anaplastic thyroid cancer (ATC) is the most lethal tumor arising from thyroid follicular epithelium. According to the TNM stage system of American Joint Committee on Cancer (AJCC), all ATC patients are classified as stage IV.^[^ ^1^ ^]^ As reported previously,^[^ ^2^ ^]^ the median survival of ATC patients is ≈5 months, and 20% of them have 1‐year overall survival. Despite significant advances in the treatment of ATC, such as dabrafenib and trametinib,^[^ ^1^ ^]^ there is no single therapeutic approach that is durably effective and resistance‐exempt, which requires multidisciplinary therapies and more translational research in novel drug targets from basic science to clinical practice.

Lenvatinib is a tyrosine kinase inhibitor targeting VEGFR1‐3, FGFR1‐4, PDGFR‐α, KIT and RET, that is approved by the US food and drug administration (FDA) for treatmenting refractory differentiated thyroid cancer (DTC), advanced renal cell carcinoma and hepatocellular carcinoma, and it is under clinical trials in various cancers.^[^ ^3^ ^]^ Based on the excellent results of the randomized, double‐blind, multicenter, phase 3 trial (SELECT) in DTC,^[^ ^4^ ^]^ lenvatinib is approved in 2015 for use in cases of locally recurrent or metastatic, progressive, radioactive iodine‐refractory DTC, which prompts clinical researchers to have high expectation for lenvatinib's efficacy in ATC. In a phase 2, single‐arm, open‐label study of lenvatinib from Japan, patients had a median progression‐free survival of 7.4 months and a median overall survival of 10.6 months, and the objective response rate among 17 ATC patients was 24%.^[^ ^5^, ^6^ ^]^ Based on the Japanese phase II results, lenvatinib was approved for ATC therapy in Japan, and in other countries it was an off‐label use option for ATC patients. Though lenvatinib shows clinical activity in a minority of studies,^[^ ^7^, ^8^ ^]^ more subsequent studies demonstrate that lenvatinib monotherapy have limited efficacy in ATC.^[^ ^9^, ^10^, ^11^, ^12^ ^]^ Furthermore, due to lack of efficacy, the International Thyroid Oncology Group discontinued an intended confirmatory phase 2 trial of lenvatinib in ATC at the interim analysis stage.^[^ ^1^ ^]^ As reported in the previous researches, the mechanisms underlying the development of lenvatinib resistance in tumor therapy vary dependent on cancer types and disease status, which are associated with cellular transformation‐related processes (epithelial‐mesenchymal transition, cancer stem cell, ferroptosis, autophagy), DNA damage response, metabolic reprogramming, and epigenetics (RNA modification and post‐translational modification) due to reactivation of the originally targeted signaling pathway, or activation of the bypass pathway and the novel tumor progression signaling pathway.^[^ ^3^, ^13^ ^]^ Some recent studies have revealed that lenvatinib resistance is associated with Nrf2,^[^ ^14^ ^]^ IRAK1/4^[^ ^15^ ^]^ and miR‐634^[^ ^16^ ^]^ in ATC, and lenvatinib efficacy is augmented by inhibition of Nrf2 or IRAK1/4, or overexpression of miR‐634. Moreover, several therapeutic approaches are reported to have synergistic antitumor effect with lenvatinib in preclinical ATC models, such as anti‐PD‐1/PD‐L1 therapy,^[^ ^17^ ^]^ vinorelbine^[^ ^18^ ^]^ and MEK inhibitors.^[^ ^19^ ^]^ Thus, it is critical to uncover the molecular mechanisms underlying lenvatinib resistance, in order to develop a strategy of combined therapy with lenvatinib for ATC.

Considering the clinical refractoriness and molecular‐landscape variety of ATC and the complex mechanism of lenvatinib resistance, it is the first time that we have built primary patient‐derived cells (PDCs) and patient‐derived xenografts (PDXs) of ATC with primary resistance to lenvatinib, by which the present study aims to identify a novel target associated with lenvatinib resistance, and to confirm the synergistic anti‐tumor effects of the combined therapy in vitro and vivo.

## 2 Results

### 2.1 Dinaciclib is Synergistically Lethal with Lenvatinib in ATC

**Figure 1:**
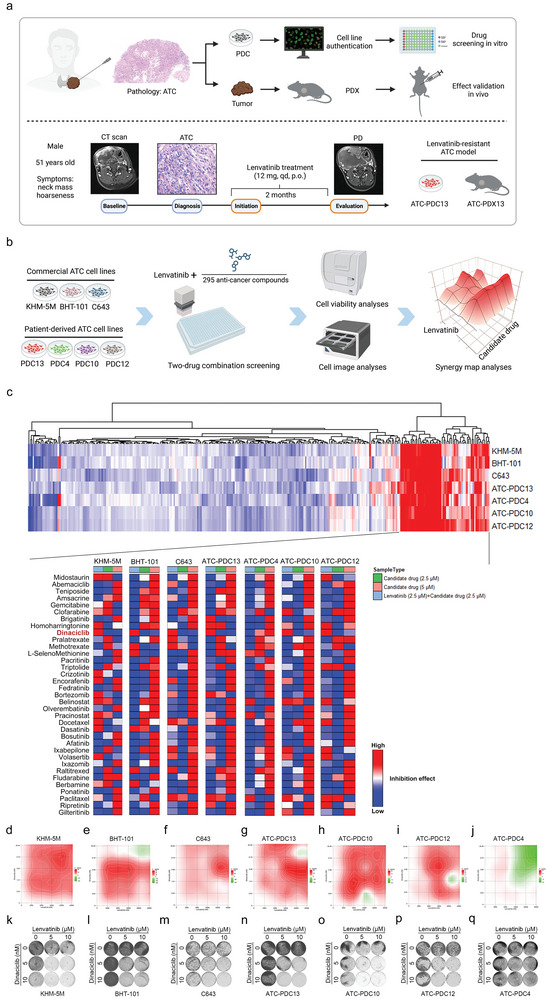
High‐throughput drug screening and synergy analyses in lenvatinib‐resistant ATC cells. a) Establishment of PDCs and PDXs from ATC patents including lenvatinib‐resistant ATC patients, Created in BioRender. Ma, B. (2024), https://BioRender.com/z49k214. b) Three commercial cell lines and four PDCs of ATC were subjected to two‐drug combination screening of lenvatinib and 295 anti‐cancer compounds, cell viability assays of CellCounting‐Lite by an Envision plate reader and synergy effect analyses by SynergyFinder, Created in BioRender. Ma, B. (2024), https://BioRender.com/z49k214. c) Heatmap analyses of inhibition rates of 295 anti‐cancer compounds combined with lenvatinib for the primary screening (the row data normalized by z‐score), the combined drug with high inhibition rates enriched in the right heatmap (above, z‐score from −3 to 3); heatmap of comparison among the selected 35 drugs combined with lenvatinib, the single 2.5 µM candidate drug and the single 5 µM candidate drug in inhibition effect for the secondary screening (below, z‐score from −1 to 1). d‐j) The Bliss independence model of SynergyFinder visualized the combined inhibition effect of lenvatinib and dinaciclib based on 6 × 6 dose matrix of cell viability assays in KHM‐5M (d), BHT‐101 (e), C643 (f), PDC13 (g), PDC10 (h), PDC12 (i) and PDC4 (j). k‐q) Cell colony formation assays of lenvatinib (0 µM, 5 µM, 10 µM) combined with dinaciclib (0, 5, and 10 nM) in KHM‐5M (k), BHT‐101 (l), C643 (m), PDC13 (n), PDC10 (o), PDC12 (p) and PDC4 (q).

We used clinical ATC samples including lenvatinib‐resistant tumors to build PDCs and PDXs as preclinical models, which were tested for sensitivity to lenvatinib and the subsequent combined drug screening with lenvatinib (**Figure** **1**a). There were five PDCs (ATC‐PDC4, ATC‐PDC7, ATC‐PDC10, ATC‐PDC12 and ATC‐PDC13) and three PDXs (ATC‐PDX10, ATC‐PDX12, and ATC‐PDX13) enrolled in this study (Figure S1a,b, Supporting Information), and they were obtained and built from patients at initial diagnosis of ATC. ATC‐PDC13 and ATC‐PDX13 were derived from a primary lenvatinib‐resistant ATC patient, who was a male of 51 years old diagnosed with ATC, presented with neck mass and hoarseness, and underwent disease progression after lenvatinib therapy (Figure 1a; Figure S1a, Supporting Information). Consistent with the limited inhibitory effect in PDCs, most ATC commercial cell lines displayed intrinsic resistance to lenvatinib in both cell viability assays and colony formation assays (Figure S1c–e, Supporting Information).

The resistant cells (IC_50_>20 µM) including three commercial cell lines (KHM‐5M, BHT‐101 and C643) and four PDCs (PDC13, PDC4, PDC10 and PDC12) were selected to perform high‐throughput drug screening (Figure 1b), in order to identify candidate drugs that have synergistic effect with lenvatinib. A panel of 295 compounds (approved anti‐cancer drugs and agents targeting tumor‐associated signaling pathways) was screened in combination with lenvatinib to explore synergy effect in the above seven cells (Figure 1b; Table S1, Supporting Information). After the primary screening of the 295 compounds, 35 compounds were selected for their potential of synergy effect with lenvatinib. As a result, of the 35 compounds, compared with treatment of dinaciclib alone or lenvatinib alone, dinaciclib repeatedly showed the significant inhibitory effect in combination with lenvatinib on ATC cells except PDC4 (Figure 1c). The Bliss independence model was used to evaluate the synergistic effect of dinaciclib and lenvatinib on cell viability, by which we observed dinaciclib at low nanomolar concentrations showed strong synergy with lenvatinib in KHM‐5M, BHT‐101, C643, PDC13, PDC10 and PDC12 (Figure 1d–i; Figure S1f–k, Supporting Information). Consistently, combination of dinaciclib and lenvatinib synergistically caused remarkable suppression of cell colony formation (Figure 1k–p). Dinaciclib and lenvatinib exerted no synergistic inhibition of cell viability and colony formation in PDC4 (Figure 1j,q; Figure S1l, Supporting Information). Dinaciclib is a potent and selective inhibitor of cyclin‐dependent kinase (CDK) that acts on CDK2, CDK5, CDK1 and CDK9, with clinical efficacy and safety in chronic lymphocytic leukemia, which is currently under phase III trial.^[^ ^20^, ^21^ ^]^ The combination regimes based on dinaciclib are carried out in multiple malignant tumors: dinaciclib and veliparib for advanced solid tumors (phase I, NCT01434316), dinaciclib and venetoclax for relapsed or refractory acute myeloid leukemia (phase I, NCT03484520), dinaciclib and pembrolizumab for advanced breast cancer (phase I, NCT01676753). The above results suggest potential significance of the combination regimen of dinaciclib and lenvatinib in ATC, which also prompts us to investigate the lenvatinib‐resistant target in terms of dinaciclib.

### 2.2 CDK2 Inhibition Sensitizes ATC to Lenvatinib

**Figure 2:**
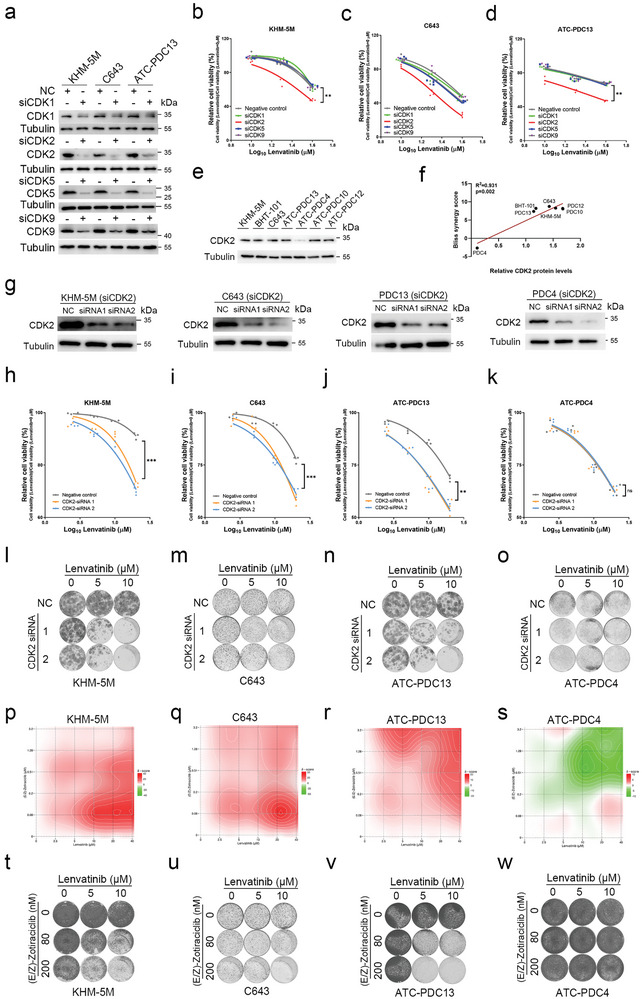
Combined inhibition effect of lenvatinib with CDK2 suppression in ATC. a) Western blot analyses of CDK1, CDK2, CDK5, CDK9 in KHM‐5M, C643 and PDC13 transfected with siRNAs against CDK1, CDK2, CDK5, CDK9 and negative control. b‐d) KHM‐5M (b), C643 (c) and PDC13 (d) were transfected with siRNAs against CDK1, CDK2, CDK5, CDK9 and negative control, followed by cell viability testing after treatment with lenvatinib (10, 20, and 40 µM). Relative cell viability is calculated as the percentage relative to the cell viability of cells treated with lenvatinib = 0 µM in each group. e) Western blot analyses of CDK2 in KHM‐5M, BHT‐101, C643, PDC13, PDC4, PDC10 and PDC12. f) Correlation of CDK2 protein expression with the synergistic effect of lenvatinib and dinaciclib in ATC cells. g) Western blot analyses of CDK2 in KHM‐5M, C643, PDC13 and PDC4 transfected with CDK2‐siRNA1, CDK2‐siRNA2 and negative control. h‐k) Relative cell viability of KHM‐5M (h), C643 (i), PDC13 (j) and PDC4 (k) transfected with CDK2‐siRNA1, CDK2‐siRNA2, negative control and treated with lenvatinib (2.5, 5, and 10 µM, 20 µM). l‐o) Cell colony formation assays of lenvatinib (0, 5, and 10 µM) combined with CDK2 knockdown by siRNA1 and siRNA2 in KHM‐5M (l), C643 (m), PDC13 (n) and PDC4 (o). p‐s) The Bliss independence model of SynergyFinder visualized the combined inhibition effect of lenvatinib and (E/Z)‐Zotiraciclib based on 6 × 6 dose matrix of cell viability assays in KHM‐5M (p), C643 (q), PDC13 (r) and PDC4 (s). t‐w) Cell colony formation assays of lenvatinib (0, 5, and 10 µM) combined with (E/Z)‐Zotiraciclib (0, 80, and 200 nM) in KHM‐5M (t), C643 (u), PDC13 (v), and PDC4 (w). ns: not significant, ^*^ *p* < 0.05, ^**^ *p* < 0.01, ^***^ *p* < 0.001.

Since dinaciclib acted as a potent and specific CDK1/2/5/9 inhibitor, we further explored the effect of intervening CDK1 or CDK2 or CDK5 or CDK9 on response to lenvatinib in vitro. CDK1, CDK2, CDK5 and CDK9 were individually knocked down in ATC cells including KHM‐5M, C643 and PDC13 by using siRNAs against CDK1, CDK2, CDK5 and CDK9 (**Figure** **2**a), which were then tested for cell viability after treatment of lenvatinib for 72 h. It was consistently observed in KHM‐5M, C643 and PDC13 that, CDK2 knockdown significantly increased the inhibitory effect of lenvatinib in comparison with the negative control, while no statistical difference was found in cell viability among the siCDK1, siCDK5, siCDK9 and negative control groups (Figure 2b–d). CDK2 protein expression was analyzed by western blot in KHM‐5M, BHT‐101, C643, PDC13, PDC4, PDC10 and PDC12 (Figure 2e). We observed a positive correlation of CDK2 protein expression with the synergistic effect of lenvatinib and dinaciclib in ATC cells, and PDC4 showed the lowest expression level of CDK2, which was associated with non‐synergistic effect of dinaciclib and lenvatinib in PDC4 (Figure 2e,f; Figure S2a, Supporting Information). We used two independent siRNAs against CDK2 to validate the impact of CDK2 knockdown on lenvatinib's effect in KHM‐5M, C643, PDC13 and PDC4, of which CDK2 inhibition combined with lenvatinib markedly reduced cell viability and colony formation except PDC4 (Figure 2g–o). The same findings were further confirmed by combination treatment of another CDK2 inhibitor‐zotiraciclib and lenvatinib (Figure 2p–w; Figure S2b–e, Supporting Information). These outcomes suggest that CDK2 suppression is synergistically lethal with lenvatinib in ATC with high CDK2 expression.

### 2.3 Clinicopathological and Biological Correlations of CDK2 in ATC

**Figure 3:**
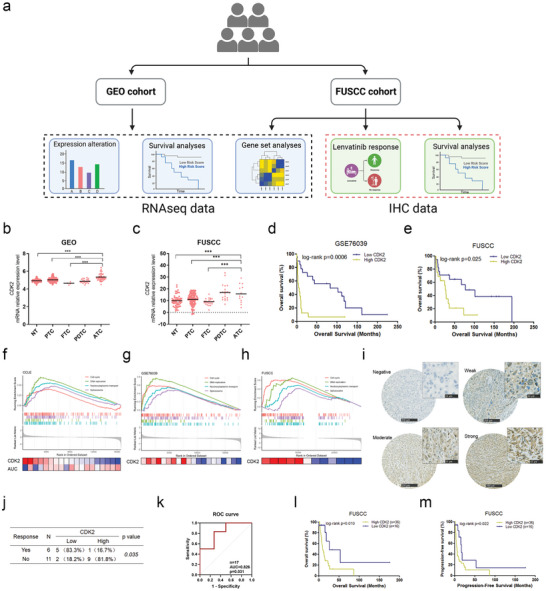
Clinicopathological associations of CDK2 in ATC. a) Workflow of CDK2 expression analyses in ATC, Created in BioRender. Ma, B. (2024), https://BioRender.com/z49k214. b,c) Comparison of CDK2 mRNA expression among normal thyroid tissues, DTC, PDTC and ATC from the GEO cohort (b) and the FUSCC cohort (c). d,e) Kaplan‐Meier curve analyses of correlations of CDK2 mRNA expression with overall survival in dedifferentiated thyroid cancer (PDTC and ATC) patients from the GEO cohort (d) and the FUSCC cohort (e). f‐h) Gene set enrichment analyses of CDK2 expression in nineteen hepatocellular carcinoma cell lines from the cancer cell line encyclopedia (CCLE) (f), the ATC patients from the GSE76039 cohort (g) and the ATC patients from the FUSCC cohort (h). i) Immunohistochemical staining of CDK2 in ATC, scale bar: 50 µm. j) Association analyses of immunohistochemical CDK2 expression with response to lenvatinib in ATC patients. k) Receiver operating characteristic (ROC) curve analyses of CDK2 expression in prediction for no response to lenvatinib treatment in ATC patients, AUC: area under curve. l‐m) Kaplan‐Meier curve of overall survival (l) and progression‐free survival (m) analyses of ATC patients with low and high immunohistochemical CDK2 expression. ^*^ *p* < 0.05, ^**^ *p* < 0.01, ^***^ *p* < 0.001.

We analyzed CDK2 expression in thyroid cancer by using the RNA sequencing data from the Gene Expression Omnibus (GEO) cohort and the Fudan University Shanghai Cancer Center (FUSCC) cohort, and the immunohistochemical (IHC) results of the FUSCC cohort, in order to explore CDK2's clinicopathological correlations in thyroid cancer (**Figure** **3**a). For patients from both the GEO cohort and the FUSCC cohort, the mRNA expression of CDK2 was significantly higher in ATC compared with normal thyroid tissues and DTCs (Figure 3b,c). The high CDK2 expression at mRNA levels was associated with decreased overall survival rate in the poorly DTC (PDTC) and ATC patients from the GEO cohort (Figure 3d) and the FUSCC cohort (Figure 3e).

Lenvatinib was approved in 2018 by the US FDA for first‐line therapy of unresectable hepatocellular carcinoma. We obtained data of response to lenvatinib treatment in nineteen hepatocellular carcinoma cell lines from the cancer therapeutics response portal (CTRP) and gene expression profiles of these cell lines available in the cancer cell line encyclopedia (CCLE). CDK2 expression showed a significant negative correlation with the area under curve (AUC) of lenvatinib response in hepatocellular carcinoma cell lines (Figure S2f, Supporting Information). The hepatocellular carcinoma cell lines with high CDK2 expression enriched biological signaling pathways including cell cycle, DNA replication, nucleocytoplasmic transportation and spliceosome, which were enriched in ATC patients with high CDK2 expression from the GSE76039 cohort and the FUSCC cohort as well (Figure 3f–h). We analyzed the correlation of CDK2 protein expression with response to lenvatinib in seventeen ATC patients from FUSCC by IHC staining of the tumor samples at initial diagnosis. As a result, the high CDK2 expression was significantly associated with no response to lenvatinib (Figure 3i,j), and the high CDK2 expression was predictive of no response to lenvatinib in ATC patients (AUC = 0.826, p = 0.031, Figure 3k). Additionally, we found that the high expression of CDK2 protein markedly correlated with decrease of overall survival rate (Figure 3l) and progression‐free survival rate (Figure 3m) in our ATC patients, suggesting CDK2 as a potential biomarker for lenvatinib therapy and prognostic predictor for ATC.

### 2.4 Combination of Lenvatinib with CDK2 Inhibition Promotes Cellular Senescence of ATC

**Figure 4:**
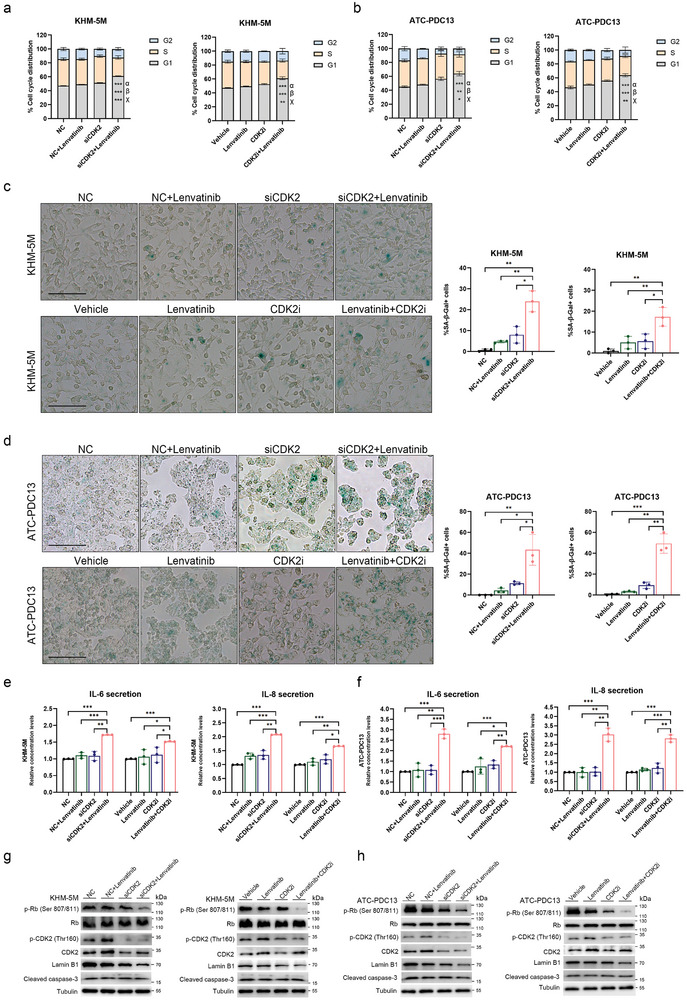
Combination effect of lenvatinib with CDK2 inhibition on cellular function in ATC. a,b) Cell cycle analyses of KHM‐5M (a) and ATC‐PDC13 (b) treated with lenvatinib or CDK2 inhibition (siCDK2: siRNA against CDK2, CDK2i: dinaciclib) or combination. Bars represent the different phases of cell distribution. α represents statistical significance in G1 phase of cell distribution between the treatment group of lenvatinib+CDK2 inhibition and the control group; β represents statistical significance in G1 phase of cell distribution between the treatment group of lenvatinib+CDK2 inhibition and the lenvatinib group, χ represents statistical significance in G1 phase of cell distribution between the treatment group of lenvatinib+CDK2 inhibition and the CDK2 inhibition group. c,d) Assessment of senescence‐associated β‐galactosidase (SA‐β‐Gal) staining cells in KHM‐5M (c) and ATC‐PDC13 (d) treated with lenvatinib or CDK2 inhibition or combination, scale bar: 100 µm. e,f) Detection of secretion of IL‐6 and IL‐8 by Enzyme‐linked Immunosorbent Assays (ELISAs) in KHM‐5M (e) and ATC‐PDC13 (f) treated with lenvatinib or CDK2 inhibition or combination. G,h) Western blot analyses of cleaved caspase‐3, Lamin B1, CDK2, phosphorylated CDK2 (Thr 160), Rb and phosphorylated Rb (Ser 807/811) in KHM‐5M (g) and ATC‐PDC13 (h) treated with lenvatinib or CDK2 inhibition or combination. All values of experiments in triplicates are expressed as means±standard deviation (SD). ^*^ *p* < 0.05, ^**^ *p* < 0.01, ^***^ *p* < 0.001.

It was initially observed that apoptosis was not significantly induced with treatment of lenvatinib alone for 72 h in KHM‐5M and PDC13. We then explored whether CDK2 inhibition promoted apoptosis of KHM‐5M and PDC13. Consequently, CDK2 inhibition by siRNAs or CDK2 inhibitor‐dinaciclib did not induce apoptosis of KHM‐5M and PDC13 in combination with lenvatinib or not (Figure S3, Supporting Information). Meanwhile, we found that combination of lenvatinib and CDK2 inhibition markedly increased the percentage of G1‐arrested cells in KHM‐5M (**Figure** **4**a) and PDC13 (Figure 4b). As shown in Figure S4a–c (Supporting Information), in the analyses of cell cycle proteins, we observed that lenvatinib induced increased expression of CDK2 protein rather than CDK2 mRNA, and caused p21 decrease at mRNA and protein levels. Expression of p16 and TP53 were not affected by lenvatinib. The above findings may suggest that cell senescence is induced by combination of lenvatinib with CDK2 inhibition. The hypothesis was confirmed by the significant increase of positive senescence‐associated β‐galactosidase (SA‐β‐gal) staining cells in siCDK2 cells exposed to lenvatinib, which were observed in cells treated with combination of dinaciclib and lenvatinib as well (Figure 4c,d). The secretory phenotype of cell senescence was also found in KHM‐5M and PDC13 treated with lenvatinib and CDK2 inhibition, with higher levels of secreted interleukin‐6 (IL‐6) and interleukin‐8 (IL‐8) (Figure 4e,f). Consistently, as shown in Figure 4g,h, western blot analyses indicated that, combination of lenvatinib with CDK2 inhibition caused decrease of the Lamin B1 protein expression and the phosphorylation levels (Ser 807/811) of Rb protein, and did not cause increase of cleaved caspase‐3.

To summarize in lenvatinib‐resistant ATC, lenvatinib led to increase of CDK2 protein expression and decrease of p21 protein expression, and reduced protein levels of p21 suppressed CDK2 inactivation. Thus, CDK2 inhibition combined with lenvatinib was responsible for significant decrease of the phosphorylation levels (Ser 807/811) of Rb protein (Figure 4g,h) and transcriptional reduction of E2F1‐responsive genes (Figure S4d, Supporting Information), which caused suppression of the Rb signaling and the subsequent G1 phase arrest and cellular senescence. To our knowledge, it is the first time that we have revealed upregulation of CDK2 protein expression by lenvatinib in lenvatinib‐resistant ATC. It is critically necessary to uncover the mechanism underlying lenvatinib‐induced increase of CDK2 protein in ATC.

### 2.5 Lenvatinib Inhibits CDK2 Degradation by Reducing Its Ubiquitination Levels

**Figure 5:**
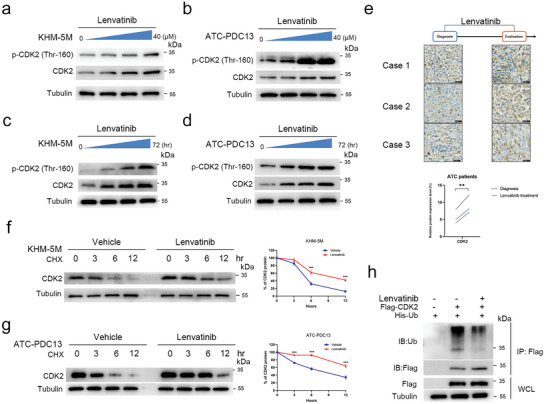
Expression alteration of CDK2 proteins in KHM‐5M and ATC‐PDC13 treated with lenvatinib. a,b) Western blot analyses of CDK2 and phosphorylated CDK2 (Thr 160) in KHM‐5M (a) and ATC‐PDC13 (b) treated with lenvatinib at the concentrations of 0, 10, 20, and 40 µM. c,d) Western blot analyses of CDK2 and phosphorylated CDK2 (Thr 160) in KHM‐5M (c) and ATC‐PDC13 (d) treated with lenvatinib for 0, 24, 48, and 72 h. e) Immunohistochemical staining of CDK2 in pre‐treated samples and post‐treated samples from lenvatinib‐resistant ATC patients, scale bar: 25 µm. f,g) KHM‐5M (f) and ATC‐PDC13 (g) were treated with or without lenvatinib (10 µM), incubated with cycloheximide (CHX, 20 µg mL^−1^) for the indicated time points. Cell lysates were immunoblotted with the CDK2 antibody and the Tubulin antibody (left). The relative abundance of CDK2 protein was normalized to the time (0 h) (right). Data of experiments in triplicates are expressed as means ± SD. h) Lenvatinib (10 µM) was used to treat KHM‐5M cells transfected with or without the flag‐CDK2 and the His‐Ubiquitin plasmids as indicated for 72 h. MG132 at the concentration of 20 µM was added to the cells 6 h before cell lysates were harvested. The ubiquitinated CDK2 was pulled down with an anti‐Flag antibody and immunoblotted with a ubiquitin antibody and an anti‐Flag antibody. Proteins of the whole cell lysates (WCL) were immunoblotted with the indicated antibodies. ^*^ *p* < 0.05, ^**^ *p* < 0.01, ^***^ *p* < 0.001.

We treated KHM‐5M and PDC13 with lenvatinib at the concentrations of 0 µM, 10 µM, 20 µM and 40 µM. It was observed that CDK2 and phosphorylated CDK2 (Thr 160) increased significantly along with the incremental concentration levels of lenvatinib (**Figure** **5**a,b). In the same way, CDK2 and phosphorylated CDK2 (Thr 160) accumulated incrementally in KHM‐5M and PDC13 with lenvatinib treatment from 0 to 72 h (Figure 5c,d). Additionally, we tested expression of CDK2 by IHC in the paired pre‐therapy and post‐therapy tumor samples from three lenvatinib‐resistant ATC patients. Consistent with the results in vitro, CDK2 expression was higher in the post‐therapy samples compared with the pre‐therapy samples (Figure 5e). The further experiments showed lenvatinib significantly attenuated degradation of CDK2 protein in KHM‐5M and PDC13 (Figure 5f,g), and lenvatinib treatment reduced the ubiquitination levels of CDK2 (Figure 5h).

### 2.6 Lenvatinib Inhibits CDK2's Ubiquitination by Attenuating CDK2's Binding with RACK1 and FBW7

**Figure 6:**
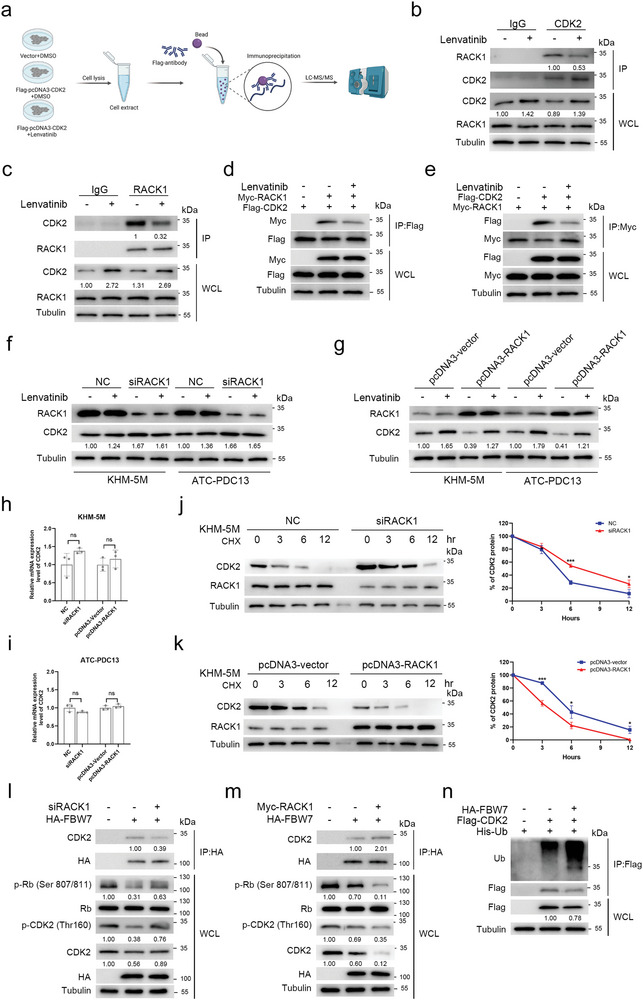
Lenvatinib caused suppression of CDK2 degradation. a) Workflow showing that lenvatinib was used to treat KHM‐5M cells transfected with or without the flag‐pCDNA3‐CDK2 plasmids, followed by co‐immunoprecipitation with anti‐Flag and protein mass spectrometry analyses, Created in BioRender. Ma, B. (2024), https://BioRender.com/z49k214. b,c) The KHM‐5M cells were treated with or without lenvatinib (10 µM) for 72 h, followed by IP with an anti‐lgG antibody and an anti‐CDK2 antibody (b) or an anti‐RACK1 antibody (c). Proteins of the pull‐down complex and the WCL were immunoblotted with a RACK1 antibody, a CDK2 antibody and a Tubulin antibody. d,e) The KHM‐5M cells transfected with or without the flag‐CDK2 and the myc‐RACK1 plasmids were treated with lenvatinib (10 µM) or DMSO for 72 h, followed by IP with an anti‐flag antibody (d) or an anti‐Myc antibody (e). Proteins of the pull‐down complex and the WCL were immunoblotted with an anti‐flag antibody, an anti‐Myc antibody and a Tubulin antibody. f,g) The KHM‐5M cells and the PDC13 cells transfected with RACK1‐siRNAs/negative control (NC) (f) or the RACK1 plasmids/vector (g) were treated with lenvatinib (10 µM) or DMSO for 72 h. Proteins of WCL were immunoblotted with the indicated antibodies. h,i) Real time‐quantitative PCR analyses of CDK2 mRNA expression in the KHM‐5M cells (h) and the PDC13 cells (i) transfected with RACK1‐siRNAs or the RACK1 plasmids. Data of experiments in triplicates are expressed as means ± SD. j,k) The KHM‐5M cells were transfected with the RACK1‐siRNAs (j) or the RACK1 plasmids (k), incubated with cycloheximide (CHX, 20 µg mL^−1^) for the indicated time points. Cell lysates were immunoblotted with the indicated antibodies (left). The relative abundance of CDK2 protein was normalized to the time (0 h) (right). Data of experiments in triplicates are expressed as means ± SD. l,m) The KHM‐5M cells were transfected with the HA‐FBW7 plasmids and the RACK1‐siRNAs (l) or the HA‐FBW7 plasmids and the myc‐RACK1 plasmids (m), followed by IP with an anti‐HA antibody. Proteins of the pull‐down complex and the WCL were immunoblotted with the indicated antibodies. n) The KHM‐5M cells were transfected with the HA‐FBW7 plasmids, the flag‐CDK2 plasmids and the His‐ubiquitin plasmids, followed by IP with an anti‐flag antibody. Proteins of the pull‐down complex and the WCL were immunoblotted with the indicated antibodies. ns: not significant, ^*^ *p* < 0.05, ^**^ *p* < 0.01, ^***^ *p* < 0.001.

To investigate how lenvatinib affected CDK2 degradation in ATC, we performed co‐immunoprecipitation assays and protein mass spectrometry analyses to identify the CDK2‐interacting target associated with lenvatinib (**Figure** **6**a). The proteomics analyses indicated that lenvatinib significantly attenuated CDK2's interaction with receptor for activated C kinase 1 (RACK1), which was a scaffolding protein involved in the ubiquitination and proteasome‐mediated degradation of proteins (Figure S5a,b, Supporting Information). CDK2's interaction with RACK1 was confirmed by the STRING interaction network (https://string‐db.org/) and the integrated interactions database (http://ophid.utoronto.ca/iid) (Figure S5c, Supporting Information). The immunoprecipitation (IP) assays confirmed that CDK2 directly bound to RACK1 in KHM‐5M, and lenvatinib treatment attenuated the endogenous combination of CDK2 with RACK1 in KHM‐5M (Figure 6b,c). To further validate CDK2's direct interaction with RACK1, we overexpressed flag‐CDK2 and myc‐RACK1 in KHM‐5M to perform the IP analyses, and the results showed that over‐expressed flag‐CDK2 bound directly to myc‐RACK1, and their binding was significantly attenuated by lenvatinib (Figure 6d,e). Then, we evaluated the impact of RACK1 on CDK2 in KHM‐5M and PDC13. As shown in Figure 6f, RACK1 knockdown increased CDK2 protein levels in KHM‐5M and PDC13 regardless of whether lenvatinib treatment was given or not. CDK2 protein levels decreased markedly in KHM‐5M and PDC13 with RACK1 overexpression while lenvatinib treatment reversed the outcomes (Figure 6g). In addition, the mRNA levels of CDK2 were not affected by RACK1 in KHM‐5M and PDC13 (Figure 6h,i), suggesting that RACK1 mediated regulation of CDK2 protein in ATC.

Considering RACK1 as a scaffolding protein associated with ubiquitination, we explored the effect of RACK1 intervention on CDK2 protein stability in KHM‐5M. It was observed that knockdown of RACK1 attenuated degradation of endogenous CDK2 protein (Figure 6j) whereas overexpression of RACK1 significantly accelerated the degradation of endogenous CDK2 (Figure 6k). We further investigated whether RACK1‐associated ubiquitination ligases or regulators mediated ubiquitination and subsequent proteasomal degradation of CDK2. Of the most common RACK1‐associated ubiquitination regulators (Cullin 2, CSN5, FBW7, HUWE1, TRIM45, VHL), FBW7 showed the direct association with RACK1 and CDK2 in ATC. In KHM‐5M overexpressing HA‐tagged FBW7, the anti‐HA IP assays indicated that, RACK1 knockdown diminished the interaction of FBW7 with CDK2 (Figure 6l), whereas RACK1 overexpression enhanced their binding (Figure 6m). Furthermore, FBW7 overexpression reduced the protein levels of CDK2, phosphorylated CDK2 (Thr 160) and phosphorylated Rb (Ser 807/811) in KHM‐5M, which were reversed by RACK1 knockdown and enhanced by RACK1 overexpression (Figure 6l,m). As shown in Figure 6n, it was confirmed by the IP assays that the ubiquitination levels of CDK2 increased significantly with FBW7 overexpression in KHM‐5M.

### 2.7 Combined Therapeutic Strategy of Lenvatinib and CDK2 Inhibitor for ATC

**Figure 7:**
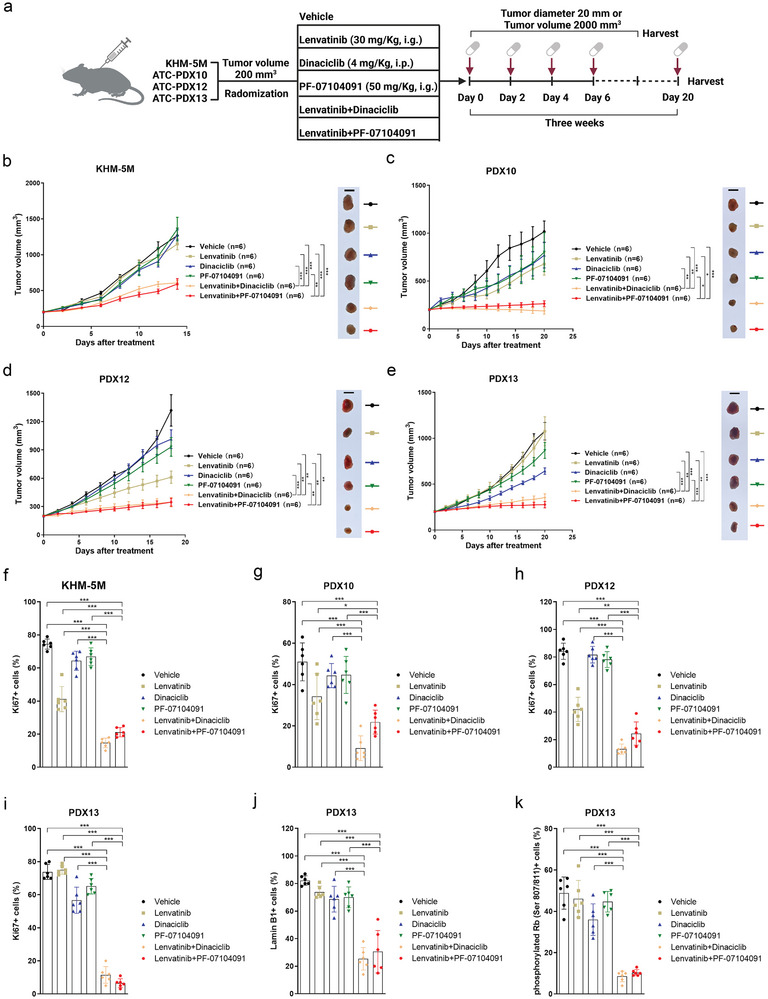
Combined treatment of lenvatinib and CDK2 inhibition in vivo. a) Subcutaneous xenograft tumors were built, and mice with xenograft tumor were randomized into six groups and received treatment with vehicle (0.5% hydroxypropylmethylcellulose, i.g.), lenvatinib (30 mg Kg^−1^, i.g.), dinaciclib (4 mg Kg^−1^, i.p.), PF‐07104091 (50 mg Kg^−1^, i.g.), lenvatinib and dinaciclib, lenvatinib and PF‐07104091 every other day for twenty‐one days, Created in BioRender. Ma, B. (2024), https://BioRender.com/z49k214. b‐e) Growth curves (left) and representative tumor mass images (right) of six groups of KHM‐5M (b), PDX10 (c), PDX12 (d) and PDX13 (e) xenografts. Scale bar: 1 cm. Data of each group (n = 6) are means±standard error of mean (SEM). f‐i) The percentage of Ki67 positive cells among the six treatment groups in KHM‐5M (f), PDX10 (g), PDX12 (h) and PDX13 (i) xenografts. Data of each group (n = 6) are means±SD. j) The percentage of Lamin B1 positive cells among the six treatment groups in PDX13 xenografts. Data of each group (n = 6) are means±SD. k) The percentage of phosphorylated Rb (Ser 807/811) positive cells among the six treatment groups in PDX13 xenografts. Data of each group (n = 6) are means±SD. ^*^ *p* < 0.05, ^**^ *p* < 0.01, ^***^ *p* < 0.001.

**Figure 8:**
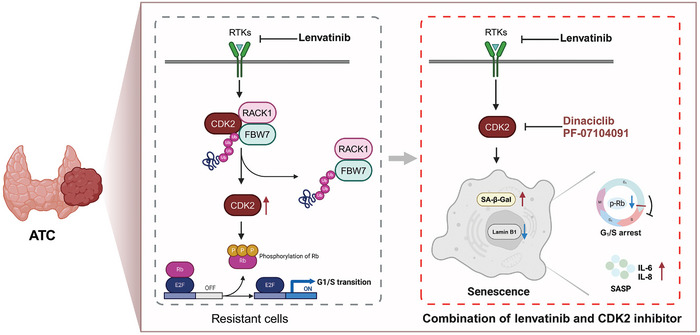
Lenvatinib‐resistant model and combination strategy of lenvatinib and CDK2 inhibition. Created in BioRender. Ma, B. (2024), https://BioRender.com/z49k214.

Next, we evaluated the efficacy of the combination of lenvatinib and CDK2 inhibitors in xenograft tumor models of ATC. Apart from dinaciclib, PF‐07104091, as a specific CDK2 inhibitor in clinical trial for solid malignant tumors, was also used for the combination therapy in vivo. Mice with xenograft tumors were treated for three weeks with the following regimens: 1) vehicle, 2) lenvatinib, 3) dinaciclib, 4) PF‐07104091, 5) the combination of lenvatinib and dinaciclib, and 6) the combination of lenvatinib and PF‐07104091 (**Figure** **7**a). The drug treatment ended if tumor size reached 2000 mm^3^ or the maximum tumor diameter reached 20 mm. As shown in Figure 7b, in the KHM‐5M xenografts, we observed that both dinaciclib combined with lenvatinib and PF‐07104091 combined with lenvatinib significantly suppressed xenograft tumor growth compared with vehicle or lenvatinib or dinaciclib or PF‐07104091 alone (Figure S6a,b, Supporting Information). To assess the effect of the combination regimen in a more valuable preclinical model, we used three PDX models from ATC patients, including the PDX13, which was obtained from a patient with primary resistance to lenvatinib. Consistent with the outcomes of the combination regimen in the KHM‐5M xenografts, we observed that xenograft tumor growth was significantly suppressed in the combination treatment group of lenvatinib and CDK2 inhibitor compared with the monotherapy group or the vehicle group for the PDX10, PDX12 and PDX13 (Figure 7c–e; Figure S6a,c–e, Supporting Information). In all xenograft tumor models, the IHC assays indicated the combination treatment caused marked downregulation of Ki67 (Figure 7f–i; Figure S6f, Supporting Information). We observed that expressions of Lamin B1 and phosphorylated Rb (Ser 807/811) were significantly inhibited in the two combination treatment groups of PDX13 (Figure 7j,k; Figure S6f, Supporting Information). Body weights of mice were maintained in the treatment groups of lenvatinib, dinaciclib, PF‐07104091 and their combination (Figure S6g–j, Supporting Information), suggesting the combination treatment was tolerated. The combination treatment prolonged the survival of tumor‐bearing mice (Figure S6k–n, Supporting Information). In summary, as shown in **Figure** **8**, we found lenvatinib inhibited CDK2 degradation via reducing CDK2's combination with the ubiquitination‐associated complex RACK1‐FBW7 in ATC. The combined therapy of lenvatinib with CDK2 inhibitor significantly induced tumor cell senescence and caused tumor growth suppression in vitro and in vivo, demonstrating an effective strategy in lenvatinib‐resistant ATC.

## 3 Discussion

Approvement of lenvatinib for refractory DTC by the US FDA increased the accessibility of lenvatinib use in ATC patients. However, it is found clinically that lenvatinib monotherapy has limited efficacy in ATC patients, and the resistance mechanism remains unclarified. In the present study, we found that CDK2 was associated with lenvatinib resistance, and CDK2 inhibition combined with lenvatinib was synergistic in anti‐tumor activity in ATC with high CDK2 expression.

CDK2 acts as a critical regulator in cell cycle, that is dysregulated in many cancer types, and emerging evidence suggest that targeting CDK2 represents a therapeutic strategy for CDK2‐dependent cancers.^[^ ^22^ ^]^ There are few studies on the role of CDK2 in ATC. According to the limited number of previous studies, CDK2 inhibition is associated with anti‐tumor effect of some anti‐cancer candidate drugs in ATC, including G1‐phase cell cycle arrest.^[^ ^23^, ^24^, ^25^ ^]^ In our study, CDK2 demonstrated a useful biomarker for ATC, which overexpressed in ATC compared with normal thyroid tissues and DTC, and showed the significant correlations with no response to lenvatinib, disease progression and decease event in ATC patients. In addition, we observed the increase of CDK2 expression in post‐therapy tumor samples from three lenvatinib‐resistant ATC patients. In cell cycle process, CDK2 is a core kinase activated by interaction with cyclin E to permit G1‐S transition. As observed in this study, CDK2 inhibition caused the G1/S transition arrest in ATC, which was augmented by combination of lenvatinib and CDK2 inhibition. Significant reduction of cell viability and colony formation by lenvatinib combined with CDK2 inhibition was not caused by apoptosis but cell cycle arrest in ATC. Cellular response of stable cell cycle arrest induced by multiple exogenous drugs is usually regarded as chemotherapy‐induced cellular senescence, including inhibitors of CDKs.^[^ ^26^ ^]^ It is well known that CDK2 plays a unique role in inhibiting oncogene/stress‐induced senescence,^[^ ^27^, ^28^, ^29^, ^30^, ^31^ ^]^ and induction of cellular senescence by targeting CDK2 has been implicated in many cancer types.^[^ ^32^, ^33^, ^34^, ^35^ ^]^ Thus, we supposed that synergistic anti‐tumor effect of lenvatinib and CDK2 inhibition was caused by their induction of cellular senescence in ATC, which was confirmed by a series of senescence‐associated experiments including the SA‐β‐gal staining, secretion of IL‐6 and IL‐8 and expression of Lamin B1 and phosphorylated Rb.

In the following analyses to figure out how lenvtinib combined with CDK2 inhibition induced increase of cell senescence, we found lenvatinib treatment not only increased CDK2 protein expression but also inhibited CDK2 inactivaiton via suppressing transcription of p21, that were the basis of synergistic effects of lenvatinib and CDK2 suppression. It is well known that p21 is the upstream molecule of CDK2, and its binding to CDK2 results in inactivation of the CDK2/cyclin E complex at the G1‐S phase DNA damage checkpoint, leading to cell arrest at the G1‐S transition for DNA repair.^[^ ^36^ ^]^ In multiple cancer types, the p21‐CDK2‐Rb signaling was found to be involved in cell cycle transition,^[^ ^37^, ^38^, ^39^ ^]^ which was targeted to induce cell senescence for cancer therapy.^[^ ^34^, ^38^, ^40^ ^]^ Digiacomo G et al reported that lenvatinib combined with CDK inhibitor induced cell senescence in hepatocarcinoma cells.^[^ ^41^ ^]^ As previously reported in ATC, p21‐cyclin/cyclin‐dependent kinase‐Rb‐E2F signaling affected cell cycle progression from the G0/G1 phase to the S phase.^[^ ^42^ ^]^ Activated CDK2‐cyclin E phosphorylate Rb to release E2F during the late G1 phase, followed by the transcription of E2F responsive genes related to cell cycle progression. Consistent with the previous findings, our study indicated that CDK2 inhibition combined with lenvatinib caused suppression of the Rb signaling and the subsequent G1 phase arrest and cellular senescence via reducing the phosphorylation levels (Ser 807/811) of Rb protein and transcriptional reduction of E2F1‐responsive genes.

To our knowledge, it is the first report in ATC that we have indicated the mechanism of lenvatinib‐mediated suppression of CDK2 degradation. In the ATC cells with synergistic inhibition effect of lenvatinib and CDK2 inhibitors, we observed that lenvatinib increased CDK2 protein expression in a dose‐ and time‐dependent manner. Mechanistically, lenvatinib markedly reduced interaction of CDK2 with RACK1, and RACK1 acted as a scaffolding protein associated with the ubiquitination and proteasome‐mediated degradation of proteins such as HIF‐1α^[^ ^43^ ^]^ and CLEC‐2.^[^ ^44^ ^]^ Consistent with the outcomes reported in the previous study,^[^ ^45^ ^]^ our further analyses also suggested the scaffolding protein combined FBW7 to form a ubiquitination‐associated complex. Lenvatinib treatment reduced combination of the RACK1‐FBW7 complex with CDK2, avoiding the ubiquitin and proteasome‐mediated degradation of CDK2.

The highlight of our study is to seek the potential combination regimen of lenvatinib and CDK2 inhibition for clinical trials in ATC. Dinaciclib, as a potent inhibitor of CDK2, is assessed in phase III trial for chronic lymphocytic leukemia,^[^ ^21^ ^]^ for advanced solid tumors in combination with veliparib (NCT01434316), for relapsed or refractory acute myeloid leukemia in combination with venetoclax (NCT03484520), phase I trial for advanced breast cancer in combination with pembrolizumab (NCT01676753). PF‐07104091, as a selective CDK2 inhibitor, is under clinical trials of multiple solid tumors (NCT04553133, NCT05262400). According to our results in vivo, combination of lenvatinib with dinaciclib or PF‐07104091 caused significant tumor growth suppression of multiple PDXs and KHM‐5M‐derived xenografts with no statistical difference in mice weight among the treated groups.

In conclusion, a lenvatinib‐resistant model was presented in our study that lenvatinib inhibited CDK2 degradation via reducing CDK2's combination with the ubiquitination‐associated complex RACK1‐FBW7 in ATC. The combined therapy of lenvatinib with CDK2 inhibitors robustly induced tumor cell senescence and caused tumor growth suppression in vitro and in vivo, and dinaciclib or PF‐07104091 may be a candidate drug combined with lenvatinib for clinical therapy of ATC patients with high expression of CDK2.

## 4 Experimental Section

### Cell Lines and Reagents

The commercial human ATC cells, KHM‐5M, BHT‐101, C643, CAL‐62, 8305C, SW1736, Hth7, TTA1, Hth83 and OCUT‐2C, were included in our study. KHM‐5M, BHT‐101, C643, CAL‐62, 8305C, Hth7, TTA1, Hth83, and OCUT‐2C were kindly provided by the Stem Cell Bank, Chinese Academy of Sciences (Shanghai, China). SW1736 was provided by the Cell Bank of University of Colorado Cancer Center (Denver, CO, USA). The cells were cultured in RPMI 1640 or DMEM with 10% FBS, glutamine, and penicillin–streptomycin (Gibco, Grand Island, NY, USA) at 37 °C and 5% CO2. KHM‐5M, BHT‐101, C643, CAL‐62, 8305C, SW1736, Hth7, TTA1, Hth83 and OCUT‐2C cells were authenticated by short tandem repeat DNA profiling by the cell line bank from which they were obtained. Lenvatinib (#S1164), dinaciclib (#S2768) and PF‐07104091 (#S9878) were purchased from Selleck Chemicals (Houston, TX, USA). A panel of 295 approved drugs or anti‐cancer agents targeting tumor‐related signaling pathways, (E/Z)‐Zotiraciclib (#HY‐15166), MG‐132 (#HY‐13259) and cycloheximide (CHX, #HY‐12320) were purchased from MedChemExpress (MCE, Newark, NJ, USA), and Table S1 (Supporting Information) contains the information of the 295 drugs.

### Human Thyroid Cancer Samples, Patient‐Derived Xenografts, and Primary ATC Cells

All of the thyroid cancer samples were obtained from thyroid cancer patients diagnosed at FUSCC. We collected the fresh core‐needle biopsy specimens or the surgical samples from ATC patients including the patients who received lenvatinib therapy from the clinical trial (NCT06195228), which were used for immunohistochemical experiments, RNA sequencing, and pre‐clinical models. The information of ATC patients was summarized in Table S2 (Supporting Information). Samples were collected, stored, and quality‐checked according to the Institutional Tissue Bank (ITB) standard procedures at FUSCC. All hematoxylin and eosin (HE) slides of the patients included in our study were subjected to evaluation by expert pathologists. We stored the fresh ATC tissue fragments in MACS tissue storage solution (#130‐100‐008, Miltenyi Biotec, Bergisch Gladbach, North Rhine‐Westphalia, Germany) and cut them into small pieces, followed by xenograft implantation and primary cell establishment within 6 h after the tumor specimens were collected from patients. The tumor pieces were implanted subcutaneously in the axilla of 4‐6‐week old female NOD‐Prkdc(em26cd52)Il2rg(em26Cd22)/Nju (NCG) mice to build the PDX model. For drug treatment validation in vivo, the tumor masses were cut and implanted into 4‐6‐week old female BALB/c nude mice once the tumor volume reached ≈ 1500 mm^3^ or the maximum diameter reached ≈ 15 mm in the third‐generation xenograft model. The hematoxylin and eosin slides of tumor masses confirmed histopathological characteristics consistent with their origination. Meanwhile, the tumor pieces were treated with digestion medium containing collagenase IV from clostridium histolyticum (1:100, 1 mg mL^−1^, #17104019, Gibco) and DMEM for primary cell extraction, followed by cell filtration with 70 µm cell strainers (#352350, Becton, Dickinson and Company, Franklin Lakes, NJ, USA). The primary ATC cells were cultured in DMEM/F‐12 (Gibco) containing 10% FBS, hydrocortisone (10 nM, #HY‐N0583, MCE), insulin (1 mg mL^−1^, #I‐5500, Sigma‐Aldrich, St. Louis, MO, USA) and penicillin‐streptomycin. All primary cells were used within 10 passages of establishment from patients, and they were routinely checked to be mycoplasma free. The primary ATC cells (PDC4, PDC7, PDC10, PDC12, and PDC13) were authenticated by cell immunofluorescent staining, whole exome sequencing, and TERT promoter mutation testing, the AE1/3, TP53, and Ki67 staining and genetic mutations indicated that the primary cells originated from ATC cells (Figure S1a,b, Supporting Information).

### In Vivo Studies

For KHM‐5M xenografts, 5 × 10^6^ cells were suspended in 200 µl mixed solution of PBS and Matrigel (50%, #354234, BD Biosciences, San Jose, CA, USA) and subsequently injected into 4‐6‐week old female BALB/c nude mice. The PDXs and KHM‐5M xenografts were treated with lenvatinib and CDK2 inhibitor for validating their combination effect in vivo. When tumor volume reached ≈ 200 mm^3^, the mice were divided into six groups at random and received treatment with vehicle (0.5% hydroxypropylmethylcellulose, i.g.), lenvatinib (30 mg Kg^−1^, i.g.), dinaciclib (4 mg Kg^−1^, i.p.), PF‐07104091 (50 mg Kg^−1^, i.g.), lenvatinib and dinaciclib, lenvatinib and PF‐07104091 every other day for twenty‐one days. The mice were sacrificed if one of the following conditions was met: 1) the treatment cycle reached 21 days, 2) the tumor volume reached 2000 mm^3^, and 3) the maximum diameter reached 20 mm. Mouse weight and tumor size were measured every other day. Tumor size was measured by digital caliper, and tumor volume was calculated based on the length and width of tumor mass: 1/2 × length × width^2^. For survival curve analysis, the deceased event was positive if the tumor volume reached or exceeded 1000 mm^3^. All animal experimental procedures were approved by the Institutional Animal Care and Use Committee of the FUSCC (IACUC‐S2022‐0342).

### High‐Throughput Drug Screening and Synergy Analyses

High‐throughput drug screening was performed at the Shanghai Institute of Biochemistry and Cell Biology, Center for Excellence in Molecular Cell Science, Chinese Academy of Sciences, as described previously.^[^ ^46^ ^]^ ATC cells were dispensed into white and clear bottom 384‐well plates (1500‐2500 cells/50 µl/well) using a Multidrop Combi reagent dispenser (Thermo Fisher Scientific, Waltham, MA, USA) and cultured overnight, followed by supplement of the 295 compounds in duplicate using a Bravo robotic workstation (Agilent Technologies, Santa Clara, CA, USA) and the D300e digital dispenser (TECAN, Mannedorf, Switzerland). Each well was treated with lenvatinib (2.5 µM) plus a combined compound (2.5 µM) for the combined treatment group, and cell wells were treated with 2.5 µM lenvatinib and 5 µM lenvatinib for the lenvatinib‐treated group. The control group was treated with DMSO. After 72 h of drug treatment, luminescent cell viability assays were performed to test cell viability using CellCounting‐Lite reagent (20 µl/well, #DD1101‐2, Vazyme Biotech, Nanjing, China), which were evaluated by an Envision plate reader (Perkin‐Elmer, Waltham, MA, USA). We calculated the average inhibition rates of two independent experiments and visualized them using z‐score normalization of the row data in heatmap by the R software (Version 3.5.1, R Foundation for Statistical Computing, Vienna, Austria). We selected the combined drugs with the average inhibition rate>3×CV (coefficient of variance) % (standard deviation/average value×100%) of DMSO‐treated cells, 2.5 µM lenvatinib‐treated cells and 5 µM lenvatinib‐treated cells for the secondary screening. For the secondary screening, cell wells were treated with 2.5 µM candidate drug and 5 µM candidate drug for the single candidate drug‐treated group. The combined treatment group was compared with the single 2.5 µM candidate drug group and the single 5 µM candidate drug group to screen out the candidate drug harboring synergistical inhibition effect with lenvatinib. Furthermore, the selected candidate drug was added in the column wells in 6 concentrations, with lenvatinib added in the row wells in 6 concentrations, in order to perform synergistic effect analyses based on a 6×6 dose matrix in duplicate. The Bliss independence model from SynergyFinder (https://synergyfinder.fimm.fi) was employed for the interactive analysis and visualization of drug combination screening data.^[^ ^47^ ^]^

### Cell Senescence Assays

Cells were cultured in 6‐well plates, and a senescence β‐galactosidase staining kit (#C0602, Beyotime Biotechnology, Shanghai, China) was used to detect SA‐β‐Gal according to the manufacture. We collected supernatant of cells, followed by IL‐6 and IL‐8 detection using the Enzyme‐linked Immunosorbent Assay (ELISA) kit of human IL‐6 (#U96‐1510E, YOBIBIO, Shanghai, China) and IL‐8 (#U96‐1513E, YOBIBIO, Shanghai, China).

### Immunohistochemical Staining and Immunofluorescent Staining

The samples of patient‐derived cancers and subcutaneous xenograft tumors were fixed in formalin and embedded in paraffin, and carcinoma regions were identified by HE sections. IHC staining was performed to detect expression of AE1/AE3 (1:100, #67306, Cell Signaling Technology, Danvers, MA, USA), TP53 (1:5000, #60283‐2‐lg, Proteintech, Chicago, IL, USA), Ki67 (1:5000, #27309‐1‐AP, Proteintech), CDK2 (1:500, #18048, Cell Signaling Technology), Lamin B1 (1:1000, #66095‐1‐lg, Proteintech) and phosphorylated Rb (Ser 807/811) (1:500, #8516, Cell Signaling Technology) according to the manufacturer's instructions. The extent of IHC staining was determined by staining intensities and the percentage of positive‐staining tumor cells in carcinoma samples.

The PDCs cultured in 384‐well plates were washed by PBS and fixed with 4% paraformaldehyde, followed by permeabilization in 0.5% Triton X‐100/PBS. The cells were washed with PBS, and then incubated in the QuickBlock blocking buffer (Beyotime) for 15 min at room temperature. Overnight incubation of the cells was performed with primary antibodies of AE1/3 (1:250, #67306, Cell Signaling Technology), TP53 (1:500, #60283‐2‐lg, Proteintech) and Ki67 (1:250, Proteintech, #27309‐1‐AP) with the QuickBlock primary antibody dilution Buffer (Beyotime) at 4 °C. Following PBS wash and incubation with the CoraLite 488‐conjugated goat anti‐rabbit IgG (1:500, #SA00013‐2, Proteintech) and the CoraLite 594‐conjugated goat anti‐mouse IgG (1:300, #SA00013‐3, Proteintech) with the QuickBlock secondary antibody dilution buffer for immunofluorescence (Beyotime) for 1 h, the cells were counterstained with DAPI staining buffer (Beyotime). An Operetta imaging system (PerkinElmer) was used to obtain the fluorescent images of the cells.

### Apoptosis, Cell Cycle, Cell Viability, and Colony Formation Assays

Following the instructions of the Annexin V‐FITC/PI kit (MULTISCIENCES Biotechnology, Hangzhou, China), after resuspending the cells in 500 µl of 1×Binding Buffer, they were incubated with 5 µl of Annexin V‐FITC and 10 µl of propidium iodide at room temperature for 5 min. Apoptotic cells were detected by flow cytometry (BD Biosciences).

Cells were stained with 1 ml DNA Staining solution and 10 µl permeabilization solution at 37 °C for 30 min, according to the instructions of the cell cycle staining kit (MULTISCIENCES Biotechnology). The cell cycle was analyzed by flow cytometry (BD Biosciences).

As described in the previous section, ATC cells were dispensed into 384‐well plates (1500‐2500 cells/50 µl/well) using a Multidrop Combi reagent dispenser (Thermo Fisher) and cultured overnight, followed by supplement of the indicated drugs in duplicate using the D300e digital dispenser (TECAN). After 72 h of drug treatment, luminescent cell viability assays were performed to test cell viability using CellCounting‐Lite reagent (Vazyme) (20 µl/well), which were evaluated by an Envision plate reader (Perkin Elmer).

ATC cells were cultured in 6‐well or 24‐well plates. After incubation with the indicated drugs for 10–14 days, cell colonies were fixed with 4% paraformaldehyde and stained with 0.1% crystal violet. Cell confluence in each well was quantified using ImageJ software.

### Western Blots

We used RIPA lysis buffer (Beyotime) with protease inhibitors (Roche, Indianapolis, IN, USA) to ATC cells. Protein concentrations were determined by the BCA method (Pierce, Thermo Fisher Scientific). The protein samples were subjected to SDS/PAGE and transferred to polyvinylidene fluoride membranes (Immobilon‐P membrane, Millipore, Bedford, MA, USA). The membranes were blocked with 5% skimmed milk in TBS plus Tween 20 at room temperature for 1 h, followed by incubation with target antibodies at 4 °C overnight. The following antibodies were used for western blot: Tubulin (1:1000, #2146, Cell Signaling Technology), CDK1 (1:1000, #77055, Cell Signaling Technology), CDK2 (1:1000, #18048, Cell Signaling Technology), CDK5 (1:1000, #2506, Cell Signaling Technology), CDK9 (1:1000, #2316, Cell Signaling Technology), p21 (1:1000, #2947, Cell Signaling Technology), p16 (1:1000, #80772, Cell Signaling Technology), TP53 (1:20000, 60283‐2‐lg, Proteintech), Lamin B1 (1:5000, 66095‐1‐lg, Proteintech), p‐CDK2 (Thr160) (1:1000, #2561, Cell Signaling Technology), Rb (1:2000, #9309, Cell Signaling Technology), p‐Rb (Ser 807/811) (1:1000, #8516, Cell Signaling Technology), cleaved caspase‐3 (1:1000, #25128‐1‐AP, Proteintech), ubiquitin (1:1000, #10201‐2‐AP, Proteintech), Flag (1:1000, #F3165, Sigma‐Aldrich), Myc (1:5000, #60003‐2‐Ig, Proteintech), HA (1:5000, #51064‐2‐AP, Proteintech), RACK1 (1:1000, #5432, Cell Signaling Technology). After incubation with HRP‐conjugated secondary antibodies for 1 h, visualization of the protein bands was achieved by an enhanced chemiluminescent chromogenic substrate using the Enhanced Chemiluminescence Plus Western Blotting Detection System (GE Healthcare, Chicago, IL, USA) and LAS‐4000EPUV mini Luminescent Image Analyzer (GE Healthcare).

### Real‐Time Quantitative Polymerase Chain Reaction (RT‐qPCR)

The Invitrogen TRIzol reagent (Thermo Fisher Scientific) was used to extract total RNA from the samples. First‐strand cDNA was synthesized using the PrimeScript Reverse Transcriptase kit (Takara, Tokyo, Japan). We utilized quantitative PCR to detect relative RNA levels on a 7900 Real‐Time PCR System with the SDS 2.3 software sequence detection system (Applied Biosystems, Thermo Fisher Scientific) using the SYBR Green (Takara) method. The primer sequences for the CDK2, p21 (CDKN1A), p16 (CDKN2A), TP53, and β‐actin (ACTB) are listed in Table S3 (Supporting Information). β‐actin as an internal control to quantify the mRNA levels of genes, and the relative levels of RNA were calculated by the comparative CT (2^−ΔΔCT^).

### Immunoprecipitation

ATC cells were lysed in IP lysis buffer (20 mM Tris‐HCl, pH 7.5, 150 mM NaCl, 1 mM EDTA, 2 mM Na_3_VO_4_, 5 mM NaF, 1% Triton X‐100, and protease and phosphatase inhibitors). The cell lysates were incubated with specific antibodies or immunoglobulin G control overnight at 4 °C, followed by incubation with protein A/G agarose beads at 4 °C for 4 h. The beads were then washed with lysis buffer and boiled in 5×SDS loading buffer for 10 min.

### Plasmid and siRNA Transfection

Complementary DNAs (cDNAs) of CDK2, RACK1 and FBW7 were amplified by RT‐PCR from U87 cells, sequenced, and then subcloned into the pcDNA3 vector (Clontech, Mountain View, CA, USA), with the tags of flag, myc and HA, respectively. The His‐ubiquitin plasmids were kindly provided by Dr. Xu Fei from FUSCC.^[^ ^48^ ^]^ These plasmids were transfected into ATC cells by Lipofectamine 3000 (Thermo Fisher Scientific). Small interference RNAs (siRNAs) against CDK1, CDK2, CDK5, CDK9 and RACK1 were transfected into ATC cells by Lipofectamin RNAiMAX (Thermo Fisher Scientific). The target sequences of the siRNAs are listed in Table S4 (Supporting Information).

### Transcriptional Profiles from the GEO Database

Raw microarray cell intensity (CEL) data of thyroid cancer, including GSE29265 (20 PTCs and 9 ATCs), GSE33630 (49 PTCs and 11 ATCs),^[^ ^49^, ^50^ ^]^ GSE53157 (15 PTCs and 5 PDTCs),^[^ ^51^ ^]^ GSE65144 (12 ATCs)^[^ ^52^ ^]^ and GSE76039 (17 PDTCs and 20 ATCs),^[^ ^53^ ^]^ were available in the GEO database (http://www.ncbi.nlm.nih.gov/geo/).^[^ ^54^, ^55^ ^]^ As described previously,^[^ ^56^ ^]^ we integrated the GSE29265 cohort, the GSE33630 cohort, the GSE53157 cohort, the GSE65144 cohort and the GSE76039 cohort into a combined GEO cohort in our study for expression analyses.

### Gene Set Enrichment Analysis (GSEA)

We performed GSEA using GSEA software obtained from the Broad Institute (http://www.broad.mit.edu/gsea),^[^ ^57^ ^]^ as described in our previous study.^[^ ^56^ ^]^ The GSEA results were visualized by enrichment map. After gene set permutations were performed 1000 times for each analysis, we used normalized enrichment score (NES) and false discovery rate (FDR) to sort the Hallmark Kyoto Encyclopedia of Genes and Genomes (KEGG) pathways enriched in each phenotype.

### Statistical Analysis

As described previously,^[^ ^58^ ^]^ we performed data collection and statistical analyses using the GraphPad Prism (Version 6.01; GraphPad Software Inc., San Diego, CA, USA), the SPSS for Windows (Version 22.0; IBM Corp., Armonk, NY, USA) and the R software (Version 3.5.1).

### Ethical Statement

This study protocol was approved by the Medical Ethics Committee of the FUSCC. All patients provided written informed consents for their specimens and information to be used for research and stored in the hospital database (approval number: 05043‐4‐2307E). All procedures performed in this study were in accordance with the ethical standards of our institutional research committee and with the 1964 Helsinki declaration and its later amendments or comparable ethical standards.

## Conflict of Interest

The authors declare no conflict of interest.

## Author Contributions

B.M., Y.S., X.D., and Y.Z. contributed equally to the study. Y.W., B.M., and Y.S. designed the study. B.M., Y.S., X.D., and Y.Z. performed experiments. T.L. provided support for experiments in vitro. B.M., X.D., D.J., and J.Z. contributed to establishment of preclinical models. B.M. and Y.S. performed data analyses. W.L., J.L., and Y.W. contributed to collection of surgical samples. M.Y. and W.X. helped prepare clinicopathological data. Q.J., D.J., N.Q., R.S., Y.W., Q.G. and J.X. provided help in enrollment of ATC patients. B.M. and Y.S. wrote the manuscript. Y.W. revised the paper. Q.J. and R.S. supervised the study. All authors have read and approved the article.

## Supporting information



Supporting Information

## Data Availability

The data that support the findings of this study are available from the corresponding author upon reasonable request.
