# Supplementary material for: Targeting CDK2 Confers Vulnerability to Lenvatinib Via Driving Senescence in Anaplastic Thyroid Cancer
Source: Adv Sci (Weinh). 2024 Dec 24;12(7):2413514. doi: 10.1002/advs.202413514 (PMC11831524; doi:10.1002/advs.202413514)
Supplement: Supplementary file 1 — Supporting Information [file ADVS-12-2413514-s001.docx]

Supporting information for

Targeting CDK2 confers vulnerability to lenvatinib via driving senescence in anaplastic thyroid cancer

Ben Ma, Youzhou Sang, Xiaoxue Du, Yanzhi Zhang, Min Yin, Weibo Xu, Wanlin Liu, Jiayi Lu, Qing Guan, Yunjun Wang, Tian Liao, Yuting Wang, Jun Xiang, Rongliang Shi, Ning Qu, Qinghai Ji, Jiwei Zhang, Dongmei Ji and Yu Wang

Correspondence to: Qinghai Ji, jiqinghai@shca.org.cn. Jiwei Zhang, joezhang@shutcm.edu.cn. Dongmei Ji, jid09@fudan.edu.cn. Yu Wang, wangyu@shca.org.cn.

**This file includes:**

Materials and Methods

Figures. S1 to S6

Tables. S1 to S4

Materials and Methods

Whole exome sequencing, TERT promoter mutation testing and RNA Sequencing

The exome DNA sequences were enriched from 0.4 μg genomic DNA using Agilent SureSelect Human All Exon V6 kit according to manufacturer’s protocol. DNA fragments were end repaired and phosphorylated, followed by A-tailing and ligation at the 3’ends with paired-end adaptors. DNA fragments with ligated adapter molecules on both ends were selectively enriched in a PCR reaction. Then, libraries hybridize with liquid phase with biotin labeled probe, and use magnetic beads with streptomycin to capture the exons of genes. Captured libraries were enriched in a PCR reaction to add index tags to prepare for sequencing. Products were purified using the AMPure XP system (Beckman Coulter, Beverly, USA), DNA concentration was measured by Qubit®3.0 Flurometer (Invitrogen, USA), libraries were analyzed for size distribution by NGS3K/Caliper and quantified by real-time PCR (3 nM). At last, DNA library were sequenced on Illumina for paired end 150 bp reads. The clustering of the index-coded samples was performed on a cBot Cluster Generation System using Illumina PE Cluster Kit (Illumina, USA) according to the manufacturer’s instructions. After that, the DNA libraries were sequenced on Illumina platform and 150 bp paired-end reads were generated.

The telomerase reverse transcriptase (TERT) promoter mutation (C228T/C250T) is determined using amplification-refractory mutation system quantitative polymerase chain reaction (ARMS-qPCR) as reported in the previous study ^1^.

Briefly, mRNA was extracted and purified from total RNA of the fresh frozen tissues using poly-T oligo-attached magnetic beads. RNA integrity was measured using the RNA Nano 6000 Assay Kit of the Bioanalyzer 2100 system (Agilent Technologies, CA, USA). Fragmentation was carried out using divalent cations under elevated temperature in First Strand Synthesis Reaction Buffer (5X). First strand cDNA was synthesized using random hexamer primer and M-MuLV Reverse Transcriptase (RNase H-). Second strand cDNA was synthesized by DNA Polymerase I and RNase H. Remaining overhangs were converted into blunt ends via exonuclease/polymerase activities. After adenylation of 3' ends of DNA fragments, Adaptor with hairpin loop structure were ligated to prepare for hybridization. In order toTo select cDNA fragments of preferentially 370~420 bp n length, the library fragments were purified with the AMPure XP system (Beckman Coulter, Beverly, USA). Then PCR was performed with Phusion High-Fidelity DNA polymerase, Universal PCR primers and Index (X) Primer. At last, PCR products were purified (AMPure XP system) and library quality was assessed on the Agilent Bioanalyzer 2100 system. The library preparations were sequenced on an Illumina Novaseq platform and 150 bp paired-end reads were generated. RNA-seq reads were aligned to the human genome reference (GRCh38/hg38) using HISAT2 (v2.0.5) ^2^. HTseq (v 2.0.2) ^3^ was utilized to count the read numbers of each gene. Normalized gene expression matrix was obtained based on the counts function of DESeq2 ^4^ (v 1.26.0) with parameter normalized = TRUE.

Reference

1. Yu PC, Tan LC, Zhu XL, Shi X, Chernikov R, Semenov A, Zhang L, Ma B, Wang Y, Zhou XY, Ji QH, Wei WJ, Wang YL. Arms-qPCR Improves Detection Sensitivity of Earlier Diagnosis of Papillary Thyroid Cancers With Worse Prognosis Determined by Coexisting BRAF V600E and Tert Promoter Mutations. Endocr Pract. 2021;27(7):698-705.
2. Kim D, Langmead B, Salzberg SL. HISAT: a fast spliced aligner with low memory requirements. Nat Methods. 2015;12(4):357-60.
3. Anders S, Pyl PT, Huber W. HTSeq--a Python framework to work with high-throughput sequencing data. Bioinformatics. 2015;31(2):166-9.
4. Love MI, Huber W, Anders S. Moderated estimation of fold change and dispersion for RNA-seq data with DESeq2. Genome Biol. 2014;15(12):550.


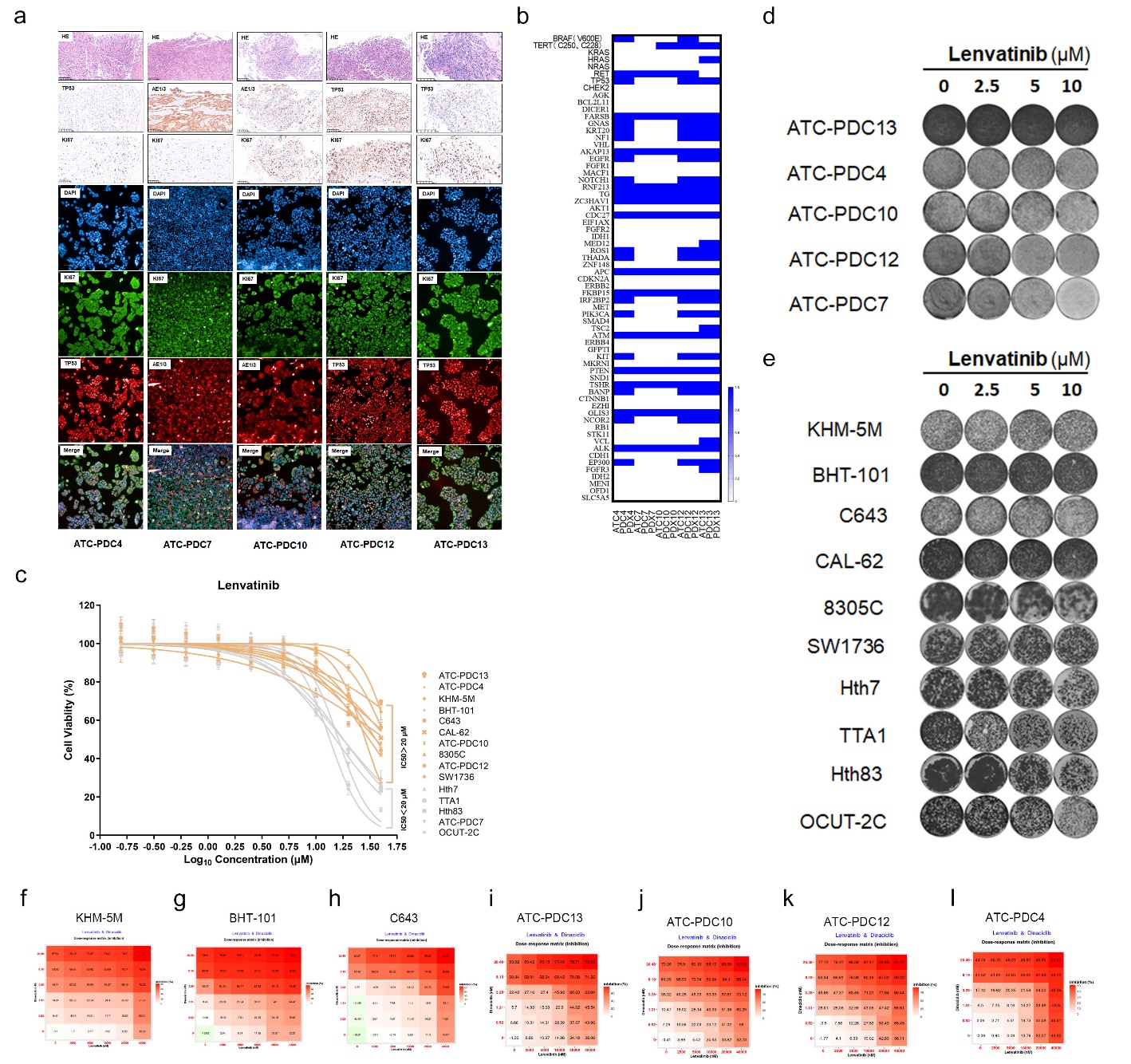


Figure S1

PDCs and commercial ATC cell lines used for screening the candidate drug with the synergistic effect with lenvatinib. **a** Immunofluorescent staining of AE1/3, TP53 and Ki67 in ATC PDCs. **b** Genetic mutations of ATC tumors, PDCs, PDXs. **c** Luminescent cell viability assays were performed to test cell viability in ATC cells using CellCounting-Lite reagent, evaluated by an Envision plate reader. **d-e** Cell colonies of PDCs (d) and commercial cell lines (e) treated with lenvatinib. **f-l** The Bliss independence model of SynergyFinder visualized the combined inhibition effect of lenvatinib and dinaciclib based on 6×6 dose matrix of cell viability assays in KHM-5M (f), BHT-101 (g), C643 (h), PDC13 (i), PDC10 (j), PDC12 (k) and PDC4 (l).


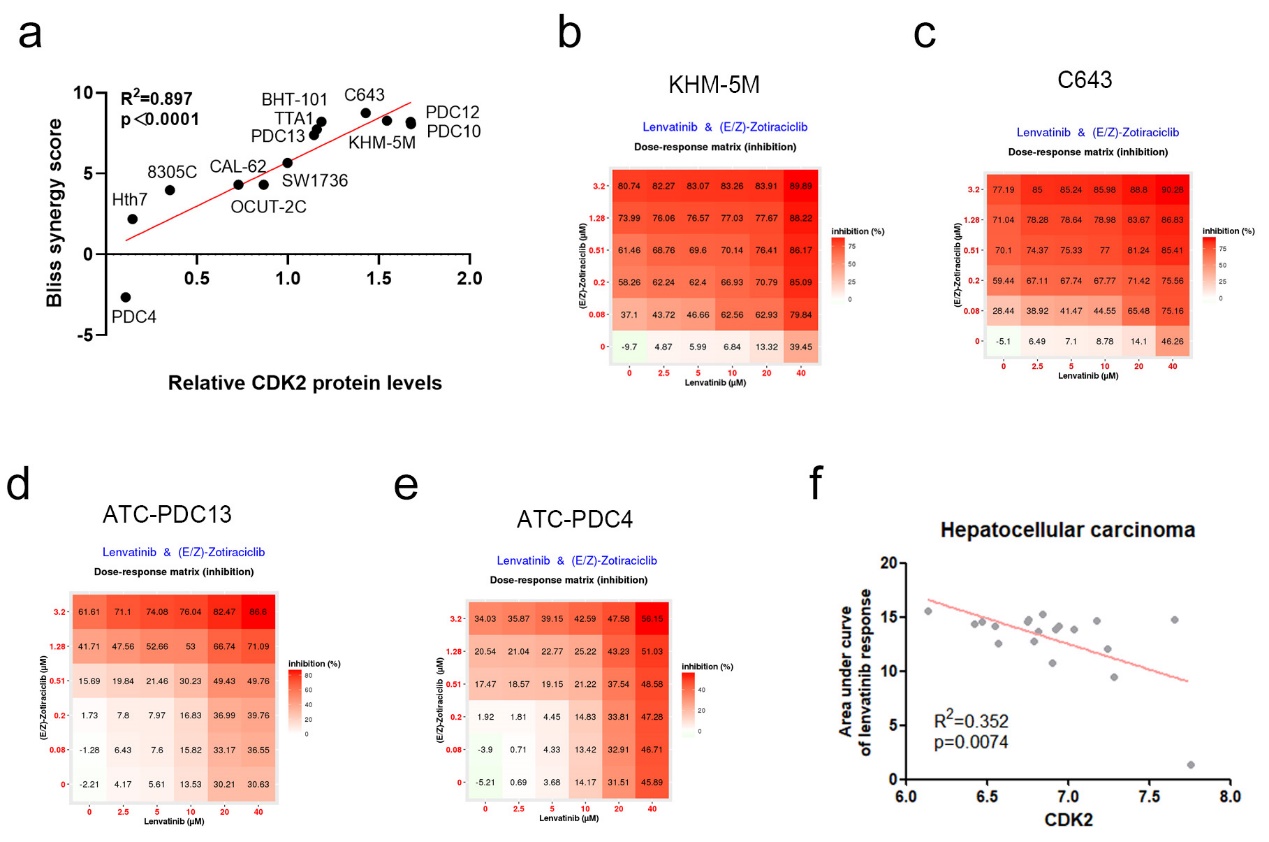


Figure S2

Targeting CDK2 correlates with lenvatinib response. **a** Correlation of CDK2 protein expression with the synergistic effect of lenvatinib and dinaciclib in ATC cells. **b-e** The Bliss independence model of SynergyFinder visualized the combined inhibition effect of lenvatinib and (E/Z)-Zotiraciclib based on 6×6 dose matrix of cell viability assays in KHM-5M (b), C643 (c), PDC13 (d) and PDC4 (e). **f** Linear correlation analyses of CDK2 expression and area under curve of lenvatinib response in hepatocellular carcinoma by using gene expression data of hepatocellular cancer cell lines from the cancer cell line encyclopedia (CCLE).


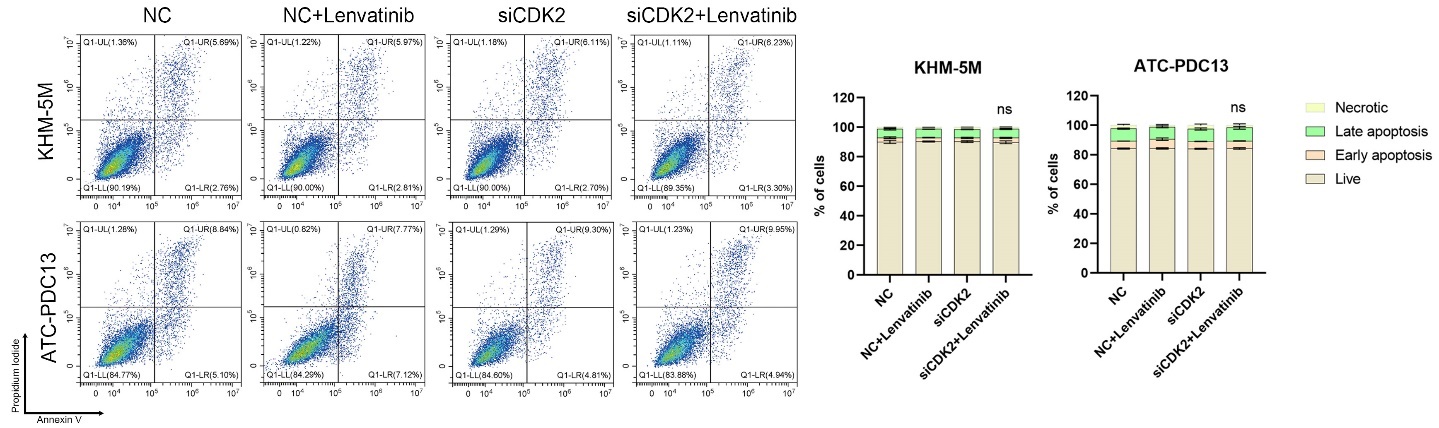


Figure S3

Annexin V/PI staining analyses of apoptotic cells induced by lenvatinib or siRNA against CDK2 or combination in KHM-5M and ATC-PDC13. ns: not significant.


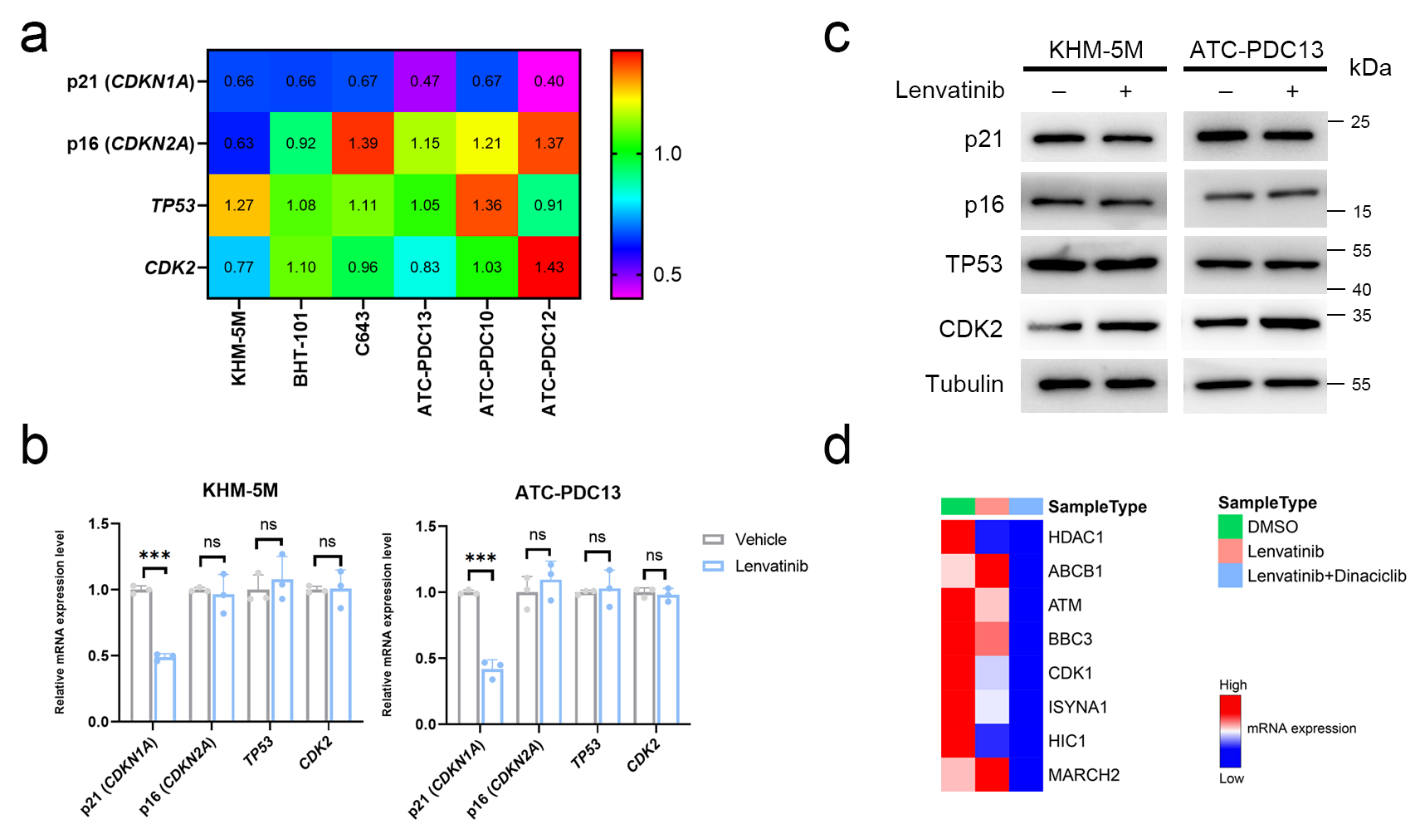


Figure S4

The impact of lenvatinib on expression of p21, p16, TP53 and CDK2. **a** The impact of lenvatinib treatment on mRNA expression of p21, p16, TP53 and CDK2 in KHM-5M, BHT-101, C643, ATC-PDC13, ATC-PDC10 and ATC-PDC12 by RNA sequencing. **b** The impact of lenvatinib treatment on mRNA expression of p21, p16, TP53 and CDK2 in KHM-5M and ATC-PDC13 by RT-qPCR. **c** The impact of lenvatinib treatment on protein expression of p21, p16, TP53 and CDK2 in KHM-5M and ATC-PDC13 by western blot. **d** The impact of lenvatinib and CDK2 inhibition on expression of E2F1 responsive genes. ns: not significant, *p < 0.05, **p < 0.01, ***p < 0.001.


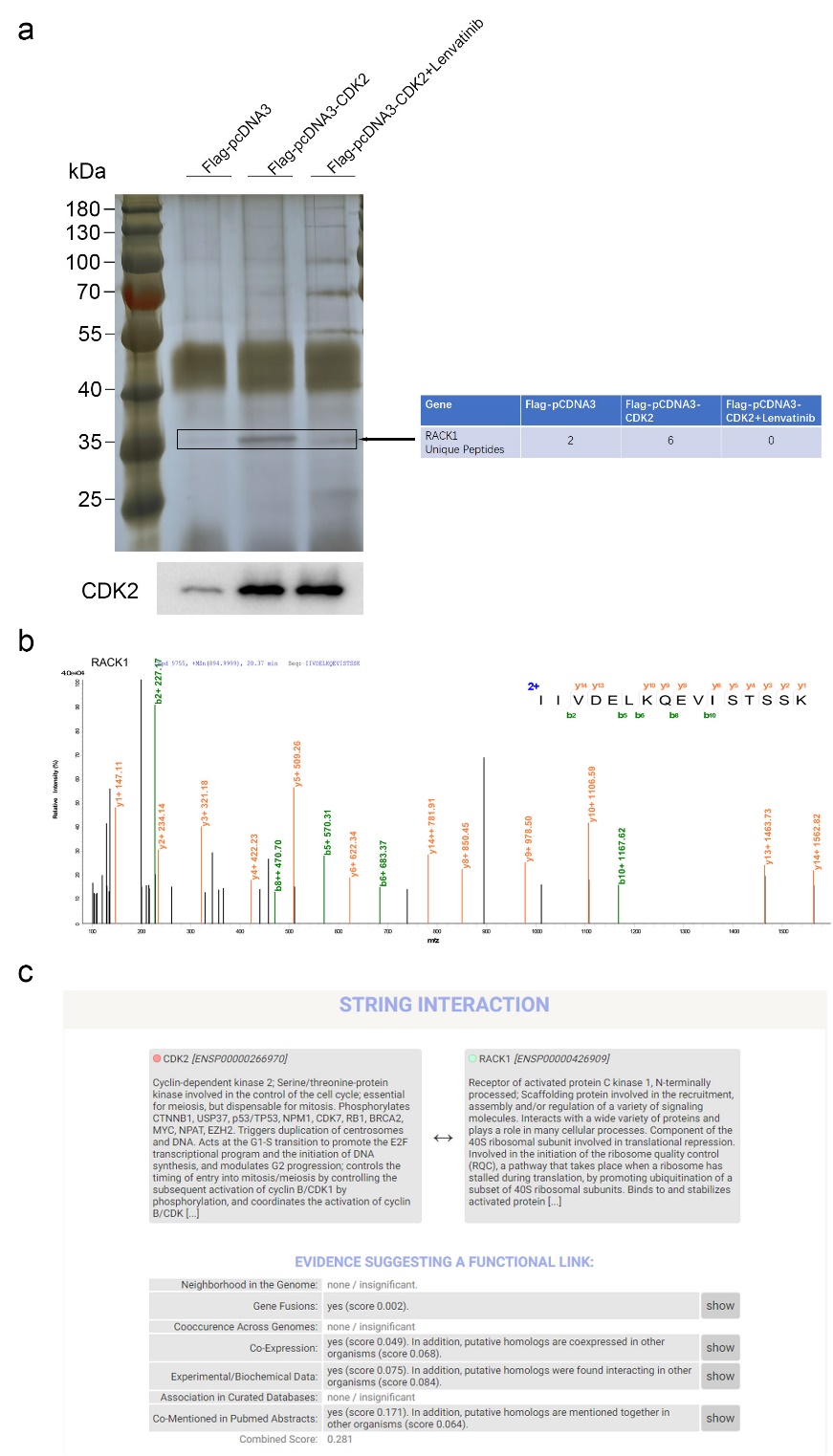


Figure S5

Interaction of RACK1 with CDK2. **a** Silver staining showing the CDK2-associated proteins affected by lenvatinib in KHM-5M. **b** Identification of RACK1 as a CDK2 binding partner by LCMS/MS analysis. Peptide sequences of RACK1 identified by LC-MS/MS analysis. **c** String interaction of RACK1 with CDK2.


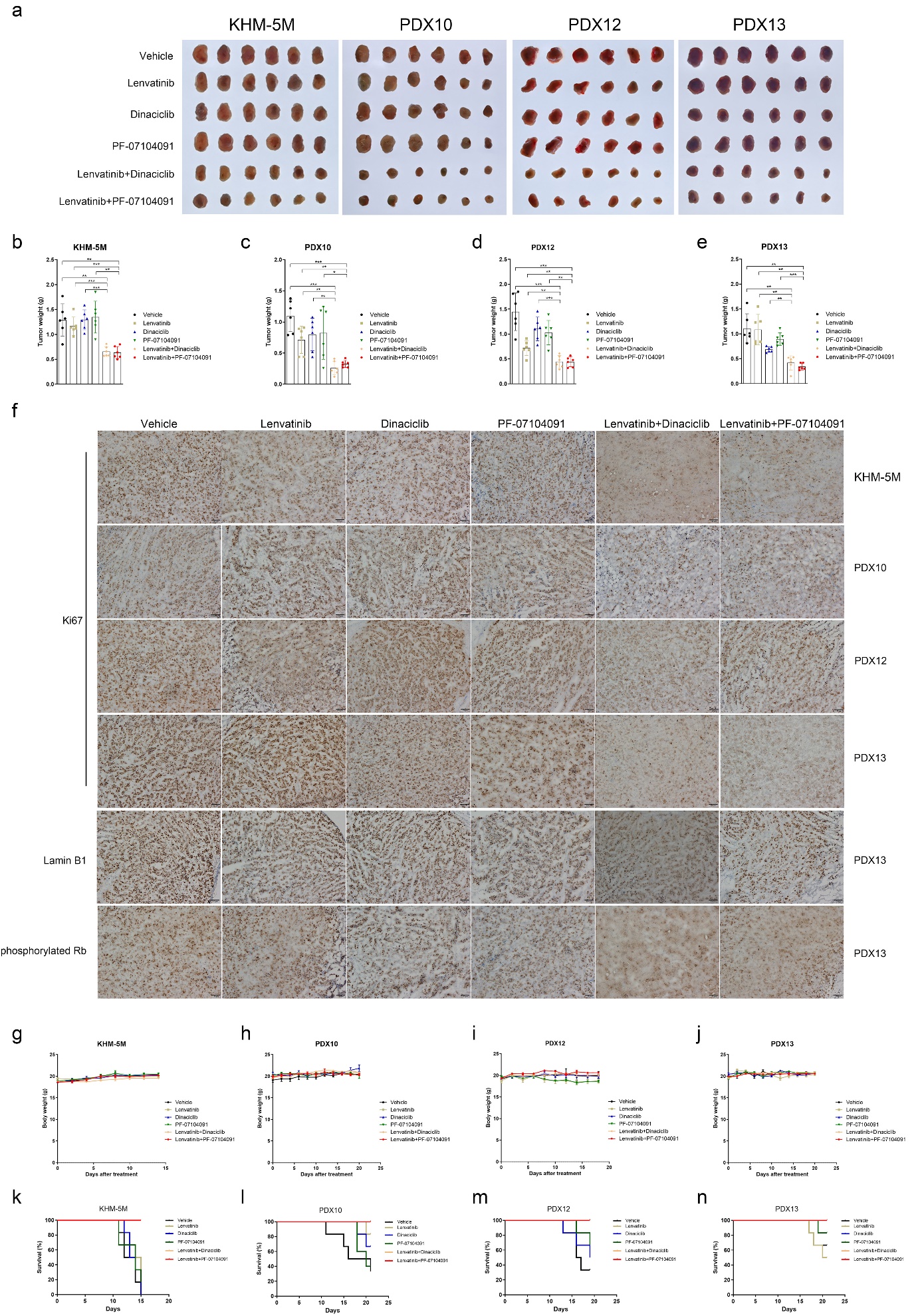


Figure S6

Results of lenvatinib combined with CDK2 inhibition in vivo. **a** Tumor mass images of six treatment groups of KHM-5M, PDX10, PDX12 and PDX13 xenografts. **b-e** Tumor weights of six groups of KHM-5M (b), PDX10 (c), PDX12 (d) and PDX13 (e) xenografts. Data of each group (n=6) are means±SEM. **f** Immunohistochemical staining (IHC) of Ki67 among six groups of KHM-5M, PDX10, PDX12 and PDX13, IHC staining of Lamin B1 and phosphorylated Rb (Ser 807/811) among six groups of PDX13. scale bar: 100 μm. **g-j** Body weights of six groups of mice bearing KHM-5M (g), PDX10 (h), PDX12 (i) and PDX13 (j) xenografts. **k-n** Survival curves of six groups of KHM-5M (k), PDX10 (l), PDX12 (m) and PDX13 (n) xenografts. *p < 0.05, **p < 0.01, ***p < 0.001.

Table S1. A panel of 295 compounds (approved anti-cancer drugs and agents targeting tumor-associated signaling pathways) was screened in combination with lenvatinib to explore synergy effect in ATC

| **Catalog Number** | **Product Name** | **CAS Number** | **M. Wt** | **Target** |
| --- | --- | --- | --- | --- |
| HY-100549 | (S)-Crizotinib | 1374356-45-2 | 450.34 | Apoptosis; DNA/RNA Synthesis |
| HY-100595 | Sodium stibogluconate | 16037-91-5 | 910.90 | Phosphatase |
| HY-10064 | Ticagrelor | 274693-27-5 | 522.57 | P2Y Receptor |
| HY-10065 | Axitinib | 319460-85-0 | 386.47 | PDGFR; VEGFR |
| HY-101467 | Trilaciclib | 1374743-00-6 | 446.55 | CDK |
| HY-101474A | Zanubrutinib | 1691249-45-2 | 471.55 | Btk |
| HY-101561 | Avapritinib | 1703793-34-3 | 498.56 | c-Kit; PDGFR |
| HY-10158 | Bosutinib | 380843-75-4 | 530.45 | Autophagy; Bcr-Abl; Src |
| HY-10162 | Olaparib | 763113-22-0 | 434.46 | Autophagy; Mitophagy; PARP |
| HY-10181 | Dasatinib | 302962-49-8 | 488.01 | Apoptosis; Autophagy; Bcr-Abl; Src |
| HY-101820 | Simotinib | 944258-89-3 | 500.95 | EGFR |
| HY-10201 | Sorafenib | 284461-73-0 | 464.83 | Apoptosis; Autophagy; Ferroptosis; FLT3; Raf; VEGFR |
| HY-10208 | Pazopanib | 444731-52-6 | 437.52 | Autophagy; c-Kit; FGFR; PDGFR; VEGFR |
| HY-10209 | Masitinib | 790299-79-5 | 498.64 | Apoptosis; c-Kit; FAK; FGFR; PDGFR; Src |
| HY-10218 | Everolimus | 159351-69-6 | 958.22 | Apoptosis; Autophagy; FKBP; mTOR |
| HY-10222 | Ixabepilone | 219989-84-1 | 506.70 | Apoptosis; Microtubule/Tubulin |
| HY-10225 | Belinostat | 866323-14-0 | 318.35 | Autophagy; HDAC |
| HY-10227 | Bortezomib | 179324-69-7 | 384.24 | Apoptosis; Autophagy; NF-κB; Proteasome |
| HY-10230 | Midostaurin | 120685-11-2 | 570.64 | Apoptosis; c-Kit; NO Synthase; PKC; VEGFR |
| HY-10255A | Sunitinib | 557795-19-4 | 398.47 | Apoptosis; Autophagy; IRE1; Mitophagy; PDGFR; VEGFR |
| HY-10260 | Vandetanib | 443913-73-3 | 475.35 | Apoptosis; Autophagy; VEGFR |
| HY-10261 | Afatinib | 850140-72-6 | 485.94 | Autophagy; EGFR |
| HY-10330 | Toceranib | 356068-94-5 | 396.46 | c-Kit; PDGFR; VEGFR |
| HY-10331 | Regorafenib | 755037-03-7 | 482.82 | Autophagy; c-Kit; FGFR; PDGFR; Raf; RET; VEGFR |
| HY-10342 | Enzastaurin | 170364-57-5 | 515.61 | Apoptosis; Autophagy; PKC |
| HY-104010 | Asciminib | 1492952-76-7 | 449.84 | Bcr-Abl |
| HY-104044 | Pamiparib | 1446261-44-4 | 298.31 | PARP |
| HY-10409 | Fedratinib | 936091-26-8 | 524.68 | Apoptosis; JAK |
| HY-10440 | Vismodegib | 879085-55-9 | 421.30 | Autophagy; Hedgehog |
| HY-10446 | Pralatrexate | 146464-95-1 | 477.47 | Antifolate; Apoptosis |
| HY-10453 | Ixazomib | 1072833-77-2 | 361.03 | Autophagy; Proteasome |
| HY-10492 | Dinaciclib | 779353-01-4 | 396.49 | Apoptosis; CDK |
| HY-10617A | Rucaparib | 283173-50-2 | 323.36 | PARP |
| HY-10619 | Niraparib | 1038915-60-4 | 320.39 | Apoptosis; PARP |
| HY-107324 | β-Elemene | 515-13-9 | 204.35 | Apoptosis |
| HY-107385 | Epristeride | 119169-78-7 | 399.57 | 5 alpha Reductase |
| HY-10820 | Pemetrexed | 137281-23-3 | 427.41 | Antifolate; Autophagy |
| HY-10821 | Raltitrexed | 112887-68-0 | 458.49 | Nucleoside Antimetabolite/Analog; Thymidylate Synthase |
| HY-108293 | Promestriene | 39219-28-8 | 328.49 | Estrogen Receptor/ERR |
| HY-109061 | Lazertinib | 1903008-80-9 | 554.64 | EGFR |
| HY-109099 | Pemigatinib | 1513857-77-6 | 487.50 | FGFR |
| HY-10977 | Tivozanib | 475108-18-0 | 454.86 | VEGFR |
| HY-10984 | Pomalidomide | 19171-19-8 | 273.24 | Apoptosis; Ligands for E3 Ligase; Molecular Glues |
| HY-10997 | Ibrutinib | 936563-96-1 | 440.50 | Btk; Ligands for Target Protein for PROTAC |
| HY-11063 | Fingolimod | 162359-55-9 | 307.47 | LPL Receptor; PAK |
| HY-112301 | Pralsetinib | 2097132-94-8 | 533.60 | RET |
| HY-112306 | Ripretinib | 1442472-39-0 | 510.36 | Apoptosis; c-Kit; FLT3; PDGFR; VEGFR |
| HY-112823 | Almonertinib | 1899921-05-1 | 525.64 | EGFR |
| HY-114277 | Sotorasib | 2296729-00-3 | 560.59 | Ras |
| HY-114370 | Selpercatinib | 2152628-33-4 | 525.60 | RET |
| HY-114778 | Fluzoparib | 1358715-18-0 | 472.40 | PARP |
| HY-12047 | Ponatinib | 943319-70-8 | 532.56 | Autophagy; Bcr-Abl; FGFR; PDGFR; Src; VEGFR |
| HY-12057 | Vemurafenib | 918504-65-1 | 489.92 | Autophagy; Raf |
| HY-12137 | Volasertib | 755038-65-4 | 618.81 | Apoptosis; Polo-like Kinase (PLK) |
| HY-12215 | Lorlatinib | 1454846-35-5 | 406.41 | ALK; Apoptosis; ROS Kinase |
| HY-12279 | Umbralisib | 1532533-67-7 | 571.55 | Casein Kinase; PI3K |
| HY-12297 | Sulfatinib | 1308672-74-3 | 480.58 | FGFR; VEGFR |
| HY-12432 | Gilteritinib | 1254053-43-4 | 552.71 | FLT3; TAM Receptor |
| HY-125840 | Belzutifan | 1672668-24-4 | 383.34 | HIF/HIF Prolyl-Hydroxylase |
| HY-12678 | Entrectinib | 1108743-60-7 | 560.64 | ALK; Autophagy; ROS Kinase; Trk Receptor |
| HY-12689 | Mitapivat | 1260075-17-9 | 450.55 | Pyruvate Kinase |
| HY-12857 | Brigatinib | 1197953-54-0 | 584.09 | ALK |
| HY-12866 | Larotrectinib | 1223403-58-4 | 428.44 | Apoptosis; Trk Receptor |
| HY-129390 | Orelabrutinib | 1655504-04-3 | 427.50 | Btk |
| HY-13001 | Quizartinib | 950769-58-1 | 560.67 | Apoptosis; Autophagy; FLT3; Ligands for Target Protein for PROTAC |
| HY-13011 | Alectinib | 1256580-46-7 | 482.62 | ALK |
| HY-13016 | Cabozantinib | 849217-68-1 | 501.51 | Apoptosis; c-Kit; c-Met/HGFR; FLT3; TAM Receptor; VEGFR |
| HY-13026 | Idelalisib | 870281-82-6 | 415.42 | Autophagy; PI3K |
| HY-13038A | Fostamatinib | 901119-35-5 | 580.46 | FLT3; Syk |
| HY-13055 | Telotristat etiprate | 1137608-69-5 | 754.15 | Tryptophan Hydroxylase |
| HY-13055A | Telotristat ethyl | 1033805-22-9 | 574.98 | Tryptophan Hydroxylase |
| HY-13272 | Dacomitinib | 1110813-31-4 | 469.94 | Apoptosis; EGFR |
| HY-13311 | Infigratinib | 872511-34-7 | 560.48 | Apoptosis; FGFR |
| HY-13322 | Pracinostat | 929016-96-6 | 358.48 | Apoptosis; HDAC |
| HY-13404 | Capmatinib | 1029712-80-8 | 412.42 | Apoptosis; c-Met/HGFR |
| HY-13407 | Gossypol | 303-45-7 | 518.55 | Bcl-2 Family |
| HY-13426 | Roxadustat | 808118-40-3 | 352.34 | Ferroptosis; HIF/HIF Prolyl-Hydroxylase |
| HY-13538 | Gemcitabine elaidate | 210829-30-4 | 527.64 | Apoptosis; Autophagy; Nucleoside Antimetabolite/Analog |
| HY-13551 | Amsacrine | 51264-14-3 | 393.46 | Autophagy; Topoisomerase |
| HY-13567 | Bendamustine | 16506-27-7 | 358.26 | Apoptosis; DNA Alkylator/Crosslinker |
| HY-135815 | Mobocertinib | 1847461-43-1 | 585.70 | EGFR |
| HY-13585 | Carmustine | 154-93-8 | 214.05 | DNA Alkylator/Crosslinker |
| HY-13593 | Chlorambucil | 305-03-3 | 304.21 | DNA Alkylator/Crosslinker |
| HY-13599 | Cladribine | 4291-63-8 | 285.69 | Adenosine Deaminase; Apoptosis |
| HY-13613 | Dutasteride | 164656-23-9 | 528.53 | 5 alpha Reductase; Apoptosis |
| HY-13635 | Finasteride | 98319-26-7 | 372.54 | 5 alpha Reductase |
| HY-13636 | Fulvestrant | 129453-61-8 | 606.77 | Apoptosis; Autophagy; Estrogen Receptor/ERR |
| HY-13669 | Lomustine | 13010-47-4 | 233.70 | Apoptosis; Autophagy; DNA Alkylator/Crosslinker |
| HY-13677 | 6-Mercaptopurine | 50-44-2 | 152.18 | Autophagy; Endogenous Metabolite; Nucleoside Antimetabolite/Analog |
| HY-13680 | Meisoindigo | 97207-47-1 | 276.29 | Apoptosis |
| HY-13683 | Mifepristone | 84371-65-3 | 429.59 | Autophagy; Glucocorticoid Receptor; NO Synthase; Progesterone Receptor |
| HY-13701 | Nelarabine | 121032-29-9 | 297.27 | Apoptosis; Nucleoside Antimetabolite/Analog |
| HY-13702 | Nilutamide | 63612-50-0 | 317.22 | Androgen Receptor |
| HY-13748 | Silibinin | 22888-70-6 | 482.44 | Autophagy; Reactive Oxygen Species |
| HY-13757A | Tamoxifen | 10540-29-1 | 371.51 | Apoptosis; Autophagy; Estrogen Receptor/ERR; HSP |
| HY-13761 | Teniposide | 29767-20-2 | 656.65 | Topoisomerase |
| HY-13771 | Ursodeoxycholic acid | 128-13-2 | 392.57 | Bile Acid Receptor; Endogenous Metabolite; FXR |
| HY-13803 | Tazemetostat | 1403254-99-8 | 572.74 | Histone Methyltransferase |
| HY-13904 | Flumatinib | 895519-90-1 | 562.59 | Bcr-Abl; c-Kit; PDGFR |
| HY-13911 | Hydroxyfasudil | 105628-72-6 | 307.37 | ROCK |
| HY-13956 | Pioglitazone | 111025-46-8 | 356.44 | Ferroptosis; PPAR |
| HY-14164 | Zileuton | 111406-87-2 | 236.29 | Ferroptosis; Lipoxygenase |
| HY-14171 | Bexarotene | 153559-49-0 | 348.48 | Autophagy; RAR/RXR |
| HY-14398 | Celecoxib | 169590-42-5 | 381.37 | COX |
| HY-14519 | Methotrexate | 59-05-2 | 454.44 | ADC Cytotoxin; Antifolate; Apoptosis; DNA/RNA Synthesis |
| HY-14566 | Donepezil | 120014-06-4 | 379.49 | Cholinesterase (ChE) |
| HY-14649 | Retinoic acid | 302-79-4 | 300.44 | Autophagy; Endogenous Metabolite; PPAR; RAR/RXR |
| HY-14652 | Tamibarotene | 94497-51-5 | 351.44 | Apoptosis; Autophagy; RAR/RXR |
| HY-14658 | Thalidomide | 50-35-1 | 258.23 | Apoptosis; Autophagy; Ligands for E3 Ligase; Molecular Glues |
| HY-14660 | Dabrafenib | 1195765-45-7 | 519.56 | Raf |
| HY-14944 | Homoharringtonine | 26833-87-4 | 545.62 | STAT |
| HY-14959 | Ulipristal | 159811-51-5 | 433.58 | Progesterone Receptor |
| HY-15025 | Sildenafil | 139755-83-2 | 474.58 | Apoptosis; Autophagy; Phosphodiesterase (PDE) |
| HY-15027 | 5-Aminosalicylic Acid | 89-57-6 | 153.14 | Endogenous Metabolite; NF-κB; PAK; PPAR |
| HY-15122 | Sinomenine | 115-53-7 | 329.39 | Apoptosis; Autophagy; NF-κB; Opioid Receptor |
| HY-15128 | 9-cis-Retinoic acid | 5300-03-8 | 300.44 | Apoptosis; Endogenous Metabolite; RAR/RXR |
| HY-15136 | Lonafarnib | 193275-84-2 | 638.82 | Autophagy; Farnesyl Transferase; Ras |
| HY-15164A | Icotinib | 610798-31-7 | 391.42 | EGFR |
| HY-15244 | Alpelisib | 1217486-61-7 | 441.47 | PI3K |
| HY-15283 | Clopidogrel | 113665-84-2 | 321.82 | P2Y Receptor |
| HY-15307 | Belumosudil | 911417-87-3 | 452.51 | ROCK |
| HY-15373 | Fenretinide | 65646-68-6 | 391.55 | Autophagy; RAR/RXR |
| HY-15388 | Tazarotene | 118292-40-3 | 351.46 | Autophagy; RAR/RXR |
| HY-15463 | Imatinib | 152459-95-5 | 493.60 | Autophagy; Bcr-Abl; c-Kit; PDGFR; SARS-CoV |
| HY-15531 | Venetoclax | 1257044-40-8 | 868.44 | Autophagy; Bcl-2 Family |
| HY-15605 | Encorafenib | 1269440-17-6 | 540.01 | Raf |
| HY-15656 | Ceritinib | 1032900-25-6 | 558.14 | ALK; IGF-1R; Insulin Receptor |
| HY-15666 | Olverembatinib | 1257628-77-5 | 532.56 | Bcr-Abl |
| HY-15771 | Tirabrutinib | 1351636-18-4 | 454.48 | Btk |
| HY-15772 | Osimertinib | 1421373-65-0 | 499.61 | EGFR |
| HY-15777 | Ribociclib | 1211441-98-3 | 434.54 | CDK |
| HY-15959 | Savolitinib | 1313725-88-0 | 345.36 | c-Met/HGFR |
| HY-16059 | Arglabin | 84692-91-1 | 246.30 | Autophagy; Farnesyl Transferase; NOD-like Receptor (NLR) |
| HY-16069 | Tucatinib | 937263-43-9 | 480.52 | EGFR |
| HY-16106 | Talazoparib | 1207456-01-6 | 380.35 | PARP |
| HY-16297A | Abemaciclib | 1231929-97-7 | 506.59 | CDK |
| HY-16322 | Minodronic acid | 180064-38-4 | 322.15 | Apoptosis; P2X Receptor |
| HY-16379 | Pacritinib | 937272-79-2 | 472.58 | FLT3; JAK |
| HY-16391 | Glasdegib | 1095173-27-5 | 374.44 | Smo |
| HY-16398 | Pipobroman | 54-91-1 | 356.05 | DNA Alkylator/Crosslinker |
| HY-16478 | Trifluridine/tipiracil hydrochloride mixture | 733030-01-8 | 435.76 | Nucleoside Antimetabolite/Analog; Thymidylate Synthase |
| HY-16500 | Tolrestat | 82964-04-3 | 357.35 | Aldose Reductase |
| HY-16503 | Treosulfan | 299-75-2 | 278.30 | DNA Alkylator/Crosslinker |
| HY-16508 | Ulipristal acetate | 126784-99-4 | 475.62 | Autophagy; Progesterone Receptor |
| HY-16513 | VAL-083 | 23261-20-3 | 146.14 | DNA Alkylator/Crosslinker |
| HY-16562 | Irinotecan | 97682-44-5 | 586.68 | Autophagy; Topoisomerase |
| HY-16582A | Sonidegib | 956697-53-3 | 485.50 | Smo |
| HY-16637 | Folic acid | 59-30-3 | 441.40 | DNA/RNA Synthesis; Endogenous Metabolite |
| HY-16749 | Pexidartinib | 1029044-16-3 | 417.81 | Apoptosis; c-Fms; c-Kit |
| HY-17026 | Gemcitabine | 95058-81-4 | 263.20 | Apoptosis; Autophagy; DNA/RNA Synthesis; Nucleoside Antimetabolite/Analog |
| HY-17044 | Duvelisib | 1201438-56-3 | 416.86 | PI3K |
| HY-17364 | Temozolomide | 85622-93-1 | 194.15 | Apoptosis; Autophagy; DNA Alkylator/Crosslinker |
| HY-17371 | Oxaliplatin | 61825-94-3 | 397.29 | Apoptosis; DNA/RNA Synthesis |
| HY-17376 | Ezetimibe | 163222-33-1 | 409.43 | Autophagy; Keap1-Nrf2 |
| HY-17386 | Rosiglitazone | 122320-73-4 | 357.43 | Autophagy; Ferroptosis; PPAR; TRP Channel |
| HY-17400 | Tegafur | 17902-23-7 | 200.17 | Nucleoside Antimetabolite/Analog |
| HY-17464 | Cilostazol | 73963-72-1 | 369.46 | Autophagy; Phosphodiesterase (PDE) |
| HY-17492 | Zafirlukast | 107753-78-6 | 575.68 | Leukotriene Receptor |
| HY-17584 | Linaclotide | 851199-59-2 | 1526.74 | Guanylate Cyclase |
| HY-17600 | Acalabrutinib | 1420477-60-6 | 465.51 | Btk |
| HY-18253 | Udenafil | 268203-93-6 | 516.66 | Phosphodiesterase (PDE) |
| HY-18300 | Filgotinib | 1206161-97-8 | 425.50 | JAK |
| HY-18690 | Enasidenib | 1446502-11-9 | 473.38 | Isocitrate Dehydrogenase (IDH) |
| HY-18708 | Erdafitinib | 1346242-81-6 | 446.54 | Apoptosis; FGFR |
| HY-18733 | Lipoic acid | 1200-22-2 | 206.33 | Endogenous Metabolite; Mitochondrial Metabolism; Reactive Oxygen Species |
| HY-18767 | Ivosidenib | 1448347-49-6 | 582.96 | Isocitrate Dehydrogenase (IDH) |
| HY-19568 | Peficitinib | 944118-01-8 | 326.39 | JAK |
| HY-19730 | Olmutinib | 1353550-13-6 | 486.59 | EGFR |
| HY-19912 | Fruquintinib | 1194506-26-7 | 393.39 | VEGFR |
| HY-32721 | Neratinib | 698387-09-6 | 557.04 | EGFR |
| HY-32735 | Triptolide | 38748-32-2 | 360.40 | Apoptosis; NF-κB |
| HY-40354 | Tofacitinib | 477600-75-2 | 312.37 | Apoptosis; JAK |
| HY-50856 | Ruxolitinib | 941678-49-5 | 306.37 | Apoptosis; Autophagy; JAK; Mitophagy |
| HY-50878 | Crizotinib | 877399-52-5 | 450.34 | ALK; Autophagy; c-Met/HGFR; ROS Kinase |
| HY-50895 | Gefitinib | 184475-35-2 | 446.90 | Apoptosis; Autophagy; EGFR |
| HY-50896 | Erlotinib | 183321-74-6 | 393.44 | Autophagy; EGFR |
| HY-50898 | Lapatinib | 231277-92-2 | 581.06 | Autophagy; EGFR; Ferroptosis |
| HY-50904 | Nintedanib | 656247-17-5 | 539.62 | FGFR; PDGFR; VEGFR |
| HY-50910 | Temsirolimus | 162635-04-3 | 1030.29 | Apoptosis; Autophagy; mTOR |
| HY-50935 | Troglitazone | 97322-87-7 | 441.54 | Apoptosis; Autophagy; Ferroptosis; PPAR |
| HY-66009 | Epalrestat | 82159-09-9 | 319.40 | Aldose Reductase |
| HY-78131B | (R)-(-)-Ibuprofen | 51146-57-7 | 206.28 | NF-κB |
| HY-90009A | Tadalafil | 171596-29-5 | 389.40 | Apoptosis; Phosphodiesterase (PDE) |
| HY-A0003 | Lenalidomide | 191732-72-6 | 259.26 | Apoptosis; Ligands for E3 Ligase; Molecular Glues |
| HY-A0004 | Decitabine | 2353-33-5 | 228.21 | Apoptosis; DNA Methyltransferase; Nucleoside Antimetabolite/Analog |
| HY-A0005 | Clofarabine | 123318-82-1 | 303.68 | Apoptosis; Autophagy; Nucleoside Antimetabolite/Analog |
| HY-A0060 | Malotilate | 59937-28-9 | 288.38 | Lipoxygenase |
| HY-A0084A | Procainamide | 51-06-9 | 235.33 | DNA Methyltransferase |
| HY-A0091A | Pargyline | 555-57-7 | 159.23 | Monoamine Oxidase |
| HY-A0168 | Regadenoson | 313348-27-5 | 390.35 | Adenosine Receptor |
| HY-B0011 | Docetaxel | 114977-28-5 | 807.88 | Apoptosis; Endogenous Metabolite; Microtubule/Tubulin |
| HY-B0012 | Pamidronic acid | 40391-99-9 | 235.07 | Wnt; β-catenin |
| HY-B0015 | Paclitaxel | 33069-62-4 | 853.91 | ADC Cytotoxin; Apoptosis; Autophagy; Microtubule/Tubulin |
| HY-B0016 | Capecitabine | 154361-50-9 | 359.35 | Apoptosis; DNA/RNA Synthesis; Nucleoside Antimetabolite/Analog |
| HY-B0021 | Doxifluridine | 3094-09-5 | 246.19 | Nucleoside Antimetabolite/Analog |
| HY-B0069 | Fludarabine | 21679-14-1 | 285.23 | Apoptosis; DNA/RNA Synthesis; Nucleoside Antimetabolite/Analog; STAT |
| HY-B0078 | Dacarbazine | 4342-03-4 | 182.18 | Apoptosis; Nucleoside Antimetabolite/Analog |
| HY-B0083 | Leflunomide | 75706-12-6 | 270.21 | Dihydroorotate Dehydrogenase; Endogenous Metabolite |
| HY-B0091 | Adapalene | 106685-40-9 | 412.52 | Apoptosis; Autophagy; RAR/RXR |
| HY-B0106 | Levetiracetam | 102767-28-2 | 170.21 | DNA Methyltransferase |
| HY-B0107 | Acitretin | 55079-83-9 | 326.43 | Apoptosis; Autophagy; RAR/RXR |
| HY-B0140 | Aminophylline | 317-34-0 | 420.43 | Adenosine Receptor; Phosphodiesterase (PDE) |
| HY-B0146 | Verteporfin | 129497-78-5 | 718.79 | Apoptosis; Autophagy; YAP |
| HY-B0150 | Nicotinamide | 98-92-0 | 122.12 | Endogenous Metabolite; Sirtuin |
| HY-B0152 | Adenine | 73-24-5 | 135.13 | DNA/RNA Synthesis; Endogenous Metabolite |
| HY-B0166 | L-Ascorbic acid | 50-81-7 | 176.12 | Apoptosis; Calcium Channel; Endogenous Metabolite; Reactive Oxygen Species |
| HY-B0181 | Altretamine | 645-05-6 | 210.28 | DNA Alkylator/Crosslinker |
| HY-B0185 | Lidocaine | 137-58-6 | 234.34 | Apoptosis; ERK; MEK; NF-κB; Sodium Channel |
| HY-B0218 | Orlistat | 96829-58-2 | 495.73 | Apoptosis; Fatty Acid Synthase (FASN) |
| HY-B0219 | Allopurinol | 315-30-0 | 136.11 | Xanthine Oxidase |
| HY-B0228 | Adenosine | 58-61-7 | 267.24 | Apoptosis; Autophagy; Endogenous Metabolite; Nucleoside Antimetabolite/Analog |
| HY-B0240 | Disulfiram | 97-77-8 | 296.54 | Aldehyde Dehydrogenase (ALDH); Interleukin Related; Pyroptosis |
| HY-B0245 | Busulfan | 55-98-1 | 246.30 | Apoptosis; DNA Alkylator/Crosslinker |
| HY-B0256 | Azathioprine | 446-86-6 | 277.26 | Apoptosis |
| HY-B0302 | Etidronic acid | 2809-21-4 | 206.03 | Apoptosis |
| HY-B0312 | Dipyridamole | 58-32-2 | 504.63 | Apoptosis; Phosphodiesterase (PDE) |
| HY-B0315 | Vitamin B12 | 68-19-9 | 1355.37 | Endogenous Metabolite |
| HY-B0351 | Taurine | 107-35-7 | 125.15 | Autophagy; Endogenous Metabolite |
| HY-B0399 | L-Carnitine | 541-15-1 | 161.20 | Endogenous Metabolite |
| HY-B0456 | Riboflavin | 83-88-5 | 376.36 | Endogenous Metabolite |
| HY-B0513 | Methylthiouracil | 56-04-2 | 142.18 | ERK; Interleukin Related; NF-κB; TNF Receptor |
| HY-B0553 | Methazolamide | 554-57-4 | 236.27 | Carbonic Anhydrase |
| HY-B0594 | Iohexol | 66108-95-0 | 821.14 | Autophagy; Mitophagy |
| HY-B0627 | Metformin | 657-24-9 | 129.16 | AMPK; Autophagy; Mitophagy |
| HY-B0639 | Amifostine | 20537-88-6 | 214.22 | HIF/HIF Prolyl-Hydroxylase; MDM-2/p53 |
| HY-B0642 | Isosorbide mononitrate | 16051-77-7 | 191.14 | Autophagy; Endogenous Metabolite |
| HY-B0660 | Eicosapentaenoic Acid | 10417-94-4 | 302.45 | Endogenous Metabolite; Histone Demethylase |
| HY-B0667 | Balsalazide | 80573-04-2 | 357.32 | Interleukin Related; STAT |
| HY-B0673 | Pirfenidone | 53179-13-8 | 185.22 | CCR; TGF-beta/Smad |
| HY-B0719 | Ingenol Mebutate | 75567-37-2 | 430.53 | PKC |
| HY-B0763 | Ibudilast | 50847-11-5 | 230.31 | Phosphodiesterase (PDE) |
| HY-B0779 | Teprenone | 6809-52-5 | 330.55 | HSP |
| HY-B0809 | Theophylline | 58-55-9 | 180.16 | Adenosine Receptor; Autophagy; Endogenous Metabolite; Phosphodiesterase (PDE) |
| HY-B1000 | Selenomethionine | 1464-42-2 | 196.11 | Autophagy; Endogenous Metabolite |
| HY-B1000A | L-SelenoMethionine | 3211-76-5 | 196.11 | Apoptosis; Endogenous Metabolite |
| HY-B1016 | Trapidil | 15421-84-8 | 205.26 | PDGFR |
| HY-B1278 | D-α-Tocopherol acetate | 58-95-7 | 472.74 | Endogenous Metabolite |
| HY-B1305 | Chloropyramine hydrochloride | 6170-42-9 | 326.26 | FAK; Histamine Receptor; VEGFR |
| HY-B1328 | Pyridoxine | 65-23-6 | 169.18 | Endogenous Metabolite; Keap1-Nrf2 |
| HY-B1334A | Perhexiline maleate | 6724-53-4 | 393.56 | Mitochondrial Metabolism |
| HY-B1391 | D-Panthenol | 81-13-0 | 205.25 | Endogenous Metabolite |
| HY-B1424 | Benzthiazide | 91-33-8 | 431.94 | Carbonic Anhydrase |
| HY-B1449 | Uridine | 58-96-8 | 244.20 | Endogenous Metabolite; Nucleoside Antimetabolite/Analog |
| HY-B1505 | Acefylline | 652-37-9 | 238.20 | Adenosine Receptor; Phosphodiesterase (PDE); Protein Arginine Deiminase |
| HY-B1508 | Vitamin K4 | 573-20-6 | 258.27 | Apoptosis; Endogenous Metabolite |
| HY-B1640 | Ethacrynic acid | 58-54-8 | 303.14 | Calcium Channel; Gutathione S-transferase; NF-κB |
| HY-B2114 | Escin | 6805-41-0 | 1131.26 | Apoptosis |
| HY-B2123 | Lactose | 63-42-3 | 342.30 | Endogenous Metabolite |
| HY-B2156 | Menaquinone-4 | 863-61-6 | 444.65 | Endogenous Metabolite |
| HY-B2167 | Docosahexaenoic acid | 6217-54-5 | 328.49 | Endogenous Metabolite |
| HY-B2194 | γ-Oryzanol | 11042-64-1 | 602.89 | DNA Methyltransferase |
| HY-B2219 | Stearic acid | 57-11-4 | 284.48 | Endogenous Metabolite |
| HY-I0960 | Uracil | 66-22-8 | 112.09 | Endogenous Metabolite |
| HY-N0019 | Daidzein | 486-66-8 | 254.24 | Endogenous Metabolite; PPAR |
| HY-N0060 | Ferulic acid | 1135-24-6 | 194.18 | Endogenous Metabolite; FGFR |
| HY-N0117 | Indirubin | 479-41-4 | 262.26 | Apoptosis |
| HY-N0124 | Dioscin | 19057-60-4 | 869.04 | Apoptosis; Autophagy |
| HY-N0139 | Troxerutin | 7085-55-4 | 742.68 | NOD-like Receptor (NLR) |
| HY-N0148 | Rutin | 153-18-4 | 610.52 | Amyloid-β; Autophagy; Endogenous Metabolite |
| HY-N0158 | Oxymatrine | 16837-52-8 | 264.36 | Apoptosis; TGF-beta/Smad |
| HY-N0159 | Paeonol | 552-41-0 | 166.17 | Autophagy; Monoamine Oxidase |
| HY-N0164 | Matrine | 519-02-8 | 248.36 | Apoptosis; Autophagy; Ferroptosis; Mitophagy; Opioid Receptor |
| HY-N0172 | Caffeic acid | 331-39-5 | 180.16 | Endogenous Metabolite; Lipoxygenase; TRP Channel |
| HY-N0180 | 18β-Glycyrrhetinic acid | 471-53-4 | 470.68 | Endogenous Metabolite |
| HY-N0198 | Nordihydroguaiaretic acid | 500-38-9 | 302.36 | Autophagy; Ferroptosis; Lipoxygenase |
| HY-N0264 | Ligustrazine | 1124-11-4 | 136.19 | Apoptosis |
| HY-N0276 | Flaconitine | 77181-26-1 | 687.77 | NF-κB |
| HY-N0322 | Cholesterol | 57-88-5 | 386.65 | Endogenous Metabolite; Estrogen Receptor/ERR |
| HY-N0375 | 18α-Glycyrrhetinic acid | 1449-05-4 | 470.68 | Apoptosis; NF-κB; Proteasome |
| HY-N0378 | D-Mannitol | 69-65-8 | 182.17 | Apoptosis; Endogenous Metabolite |
| HY-N0383 | Lappaconitine | 32854-75-4 | 584.70 | P2X Receptor |
| HY-N0390 | L-Glutamine | 56-85-9 | 146.14 | Endogenous Metabolite; Ferroptosis; mGluR |
| HY-N0455 | L-Arginine | 74-79-3 | 174.20 | Endogenous Metabolite; NO Synthase |
| HY-N0480 | Reserpine | 50-55-5 | 608.68 | Autophagy; Monoamine Transporter |
| HY-N0504 | Lovastatin | 75330-75-5 | 404.54 | Autophagy; Ferroptosis; HMG-CoA Reductase (HMGCR) |
| HY-N0510 | Aristolochic acid A | 313-67-7 | 341.27 | NF-κB |
| HY-N0523 | Gallic acid | 149-91-7 | 170.12 | Apoptosis; COX; Endogenous Metabolite; Ferroptosis; Reactive Oxygen Species |
| HY-N0538 | Xylitol | 87-99-0 | 152.15 | Autophagy; Endogenous Metabolite |
| HY-N0543 | Allantoin | 97-59-6 | 158.12 | Endogenous Metabolite; Imidazoline Receptor |
| HY-N0623 | L-Tryptophan | 73-22-3 | 204.23 | Endogenous Metabolite |
| HY-N0650 | L-Serine | 56-45-1 | 105.09 | Endogenous Metabolite |
| HY-N0697 | Crocin | 42553-65-1 | 976.96 | Endogenous Metabolite |
| HY-N0714 | Berbamine | 478-61-5 | 608.72 | Autophagy; NF-κB |
| HY-N0783 | Eupatilin | 22368-21-4 | 344.32 | Autophagy; PPAR |
| HY-N0865 | Ingenol | 30220-46-3 | 348.43 | PKC |
| HY-N1373 | Sophoridine | 6882-68-4 | 248.36 | Apoptosis |
| HY-N1404 | Sodium aescinate | 20977-05-3 | 1123.21 | NF-κB |
| HY-N1446 | Oleic acid | 112-80-1 | 282.46 | Apoptosis; Endogenous Metabolite; Na+/K+ ATPase |
| HY-N2591 | Isocorydine | 475-67-2 | 341.40 | Endogenous Metabolite |
| HY-N7075 | Inulin | 9005-80-5 | N/A | Endogenous Metabolite |
| HY-N7122 | Thymopentin | 69558-55-0 | 679.77 | Endogenous Metabolite |
| HY-N7137 | Norgestrel | 6533-00-2 | 312.45 | Reactive Oxygen Species |
| HY-W010388 | Creatine | 57-00-1 | 131.14 | Endogenous Metabolite |
| HY-W015883 | Fumaric acid | 110-17-8 | 116.07 | Endogenous Metabolite |
| HY-Y0319G | Magnesium acetate tetrahydrate | 16674-78-5 | 214.45 | Endogenous Metabolite |

Table S2. The information of the ATC patients enrolled in this study.

| ID | Gender | Age | CDK2 | Disease  progression | PFS (months) | Death | OS (months) |
| --- | --- | --- | --- | --- | --- | --- | --- |
| Case 1 | Female | 65 | Low | 0 | 176.13 | 0 | 176.13 |
| Case 2 | Female | 74 | Low | 1 | 15.47 | 1 | 15.47 |
| Case 3 | Female | 70 | High | 1 | 5.03 | 1 | 7.76 |
| Case 4 | Female | 63 | High | 1 | 2.13 | 1 | 2.13 |
| Case 5 | Male | 65 | High | 1 | 5.67 | 1 | 5.67 |
| Case 6 | Male | 52 | Low | 0 | 5.13 | 0 | 5.13 |
| Case 7 | Male | 67 | High | 1 | 86.83 | 1 | 86.83 |
| Case 8 | Male | 59 | High | 1 | 7.1 | 1 | 7.1 |
| Case 9 | Male | 68 | High | 1 | 2.63 | 1 | 2.63 |
| Case 10 | Male | 50 | High | 0 | 2.43 | 0 | 2.43 |
| Case 11 | Female | 63 | Low | 1 | 53.67 | 1 | 53.67 |
| Case 12 | Male | 51 | High | 1 | 27 | 1 | 27 |
| Case 13 | Male | 61 | High | 1 | 6.93 | 1 | 6.93 |
| Case 14 | Female | 48 | High | 1 | 3.033 | 1 | 8.26 |
| Case 15 | Male | 59 | High | 1 | 3.833 | 1 | 3.833 |
| Case 16 | Male | 64 | High | 1 | 6.3 | 1 | 9.68 |
| Case 17 | Female | 69 | Low | 1 | 0.4 | 1 | 0.4 |
| Case 18 | Male | 48 | High | 1 | 3.07 | 1 | 6.13 |
| Case 19 | Male | 86 | High | 1 | 4.8 | 1 | 4.8 |
| Case 20 | Male | 65 | High | 1 | 2.7 | 1 | 2.7 |
| Case 21 | Female | 64 | High | 0 | 8.53 | 0 | 8.53 |
| Case 22 | Female | 58 | Low | 1 | 8 | 1 | 26.34 |
| Case 23 | Male | 62 | High | 0 | 13.17 | 0 | 13.17 |
| Case 24 | Female | 75 | Low | 0 | 5.67 | 0 | 33.7 |
| Case 25 | Male | 63 | High | 1 | 10.37 | 1 | 10.37 |
| Case 26 | Male | 66 | High | 0 | 26.43 | 0 | 26.43 |
| Case 27 | Male | 62 | Low | 1 | 10.9 | 0 | 10.9 |
| Case 28 | Female | 66 | Low | 0 | 8 | 0 | 8 |
| Case 29 | Male | 70 | High | 1 | 4.53 | 1 | 4.53 |
| Case 30 | Female | 34 | Low | 1 | 18.9 | 1 | 18.9 |
| Case 31 | Female | 74 | High | 0 | 35.2 | 0 | 35.2 |
| Case 32 | Male | 46 | Low | 1 | 17.03 | 0 | 17.03 |
| Case 33 | Male | 69 | High | 1 | 22.97 | 1 | 22.97 |
| Case 34 | Male | 71 | High | 1 | 11.83 | 1 | 11.83 |
| Case 35 | Male | 50 | High | 1 | 24.37 | 1 | 24.37 |
| Case 36 | Female | 50 | High | 1 | 6.77 | 1 | 6.77 |
| Case 37 | Male | 53 | High | 1 | 3.47 | 1 | 3.47 |
| Case 38 | Female | 73 | High | 1 | 16.47 | 0 | 16.47 |
| Case 39 | Male | 56 | High | 1 | 9.73 | 1 | 9.73 |
| Case 40 | Male | 51 | High | 1 | 1.97 | 1 | 15.93 |
| Case 41 | Female | 70 | High | 1 | 3.97 | 1 | 13.56 |
| Case 42 | Male | 51 | High | 1 | 2.2 | 0 | 12.92 |
| Case 43 | Male | 54 | High | 1 | 3.7 | 1 | 13.1 |
| Case 44 | Female | 78 | Low | 0 | 13.73 | 0 | 13.73 |
| Case 45 | Male | 68 | Low | 0 | 6.73 | 0 | 6.73 |
| Case 46 | Male | 66 | High | 1 | 6 | 1 | 14.74 |
| Case 47 | Female | 68 | Low | 0 | 2 | 0 | 2 |
| Case 48 | Male | 84 | High | 0 | 1 | 0 | 1 |
| Case 49 | Male | 65 | Low | 0 | 4.94 | 0 | 4.94 |
| Case 50 | Female | 34 | High | 1 | 4.66 | 0 | 4.66 |
| Case 51 | Female | 50 | Low | 0 | 5.5 | 0 | 5.5 |
| Case 52 | Female | 67 | High | 0 | 1.6 | 0 | 1.6 |

Table S3. The primer sequences for the CDK2, p21 (CDKN1A), p16 (CDKN2A), TP53 and β-actin

| **mRNA ID** | **NCBI Gene ID** | **Target Seq** |
| --- | --- | --- |
| CDK2-forward primer | 1017 (Homo sapiens) | CCAGGAGTTACTTCTATGCCTGA |
| CDK2-reverse primer | 1017 (Homo sapiens) | TTCATCCAGGGGAGGTACAAC |
| p21 (CDKN1A)-forward primer | 1026 (Homo sapiens) | TGTCCGTCAGAACCCATGC |
| p21 (CDKN1A)-reverse primer | 1026 (Homo sapiens) | AAAGTCGAAGTTCCATCGCTC |
| p16 (CDKN2A)-forward primer | 1029 (Homo sapiens) | GGGTTTTCGTGGTTCACATCC |
| p16 (CDKN2A)-reverse primer | 1029 (Homo sapiens) | CTAGACGCTGGCTCCTCAGTA |
| TP53-forward primer | 7157 (Homo sapiens) | CAGCACATGACGGAGGTTGT |
| TP53-reverse primer | 7157 (Homo sapiens) | TCATCCAAATACTCCACACGC |
| β-actin (ACTB)-forward primer | 60 (Homo sapiens) | CATGTACGTTGCTATCCAGGC |
| β-actin (ACTB)-reverse primer | 60 (Homo sapiens) | CTCCTTAATGTCACGCACGAT |

Table S4. Small interference RNAs (siRNAs) against CDK1, CDK2, CDK5, CDK9 and RACK1.

| **siRNA** | **NCBI Gene ID** | **Target Seq** |
| --- | --- | --- |
| CDK1-siRNA 1 | 983 (Homo sapiens) | GGAACTTCGTCATCCAAAT |
| CDK1-siRNA 2 | 983 (Homo sapiens) | GGTTATATCTCATCTTTGA |
| CDK1-siRNA 3 | 983 (Homo sapiens) | GTACTGCAATTCGGGAAAT |
| CDK2-siRNA 1 | 1017 (Homo sapiens) | GCACCAAGATCTCAAGAAA |
| CDK2-siRNA 2 | 1017 (Homo sapiens) | GGATGTGACCAAGCCAGTA |
| CDK2-siRNA 3 | 1017 (Homo sapiens) | GAGTCCCTGTTCGTACTTA |
| CDK5-siRNA 1 | 1020 (Homo sapiens) | CCTCGATCCTGAGATTGTA |
| CDK5-siRNA 2 | 1020 (Homo sapiens) | CCACAACATCCCTGGTGAA |
| CDK5-siRNA 3 | 1020 (Homo sapiens) | GCGACAAGAAGCTGACTTT |
| CDK9-siRNA 1 | 1025 (Homo sapiens) | GGCCAAACGTGGACAACTA |
| CDK9-siRNA 2 | 1025 (Homo sapiens) | GAAGGCTGCTAATGTGCTT |
| CDK9-siRNA 3 | 1025 (Homo sapiens) | GAAGGCTGCTAATGTGCTT |
| RACK1-siRNA 1 | 10399 (Homo sapiens) | GACCAACTATGGAATTCCA |
| RACK1-siRNA 2 | 10399 (Homo sapiens) | GTCTCTGGATCTCGAGATA |
| RACK1-siRNA 3 | 10399 (Homo sapiens) | CCATCATCATGTGGAAACT |
